# Inhibition of HDAC1/2 Along with TRAP1 Causes Synthetic Lethality in Glioblastoma Model Systems

**DOI:** 10.3390/cells9071661

**Published:** 2020-07-10

**Authors:** Trang T. T. Nguyen, Yiru Zhang, Enyuan Shang, Chang Shu, Catarina M. Quinzii, Mike-Andrew Westhoff, Georg Karpel-Massler, Markus D. Siegelin

**Affiliations:** 1 Department of Pathology and Cell Biology, Columbia University Medical Center, New York, NY 10032, USA; tn2387@cumc.columbia.edu (T.T.T.N.); yiruzh@gmail.com (Y.Z.); cs485@cumc.columbia.edu (C.S.); 2 Department of Biological Sciences, Bronx Community College, City University of New York, Bronx, NY 10453, USA; enyuan.shang@bcc.cuny.edu; 3 Department of Neurology, Columbia University Medical Center, New York, NY 10032, USA; cmq2101@cumc.columbia.edu; 4 Department of Pediatrics and Adolescent Medicine, Ulm University Medical Center, 89081 Ulm, Germany; andrew.westhoff@uniklinik-ulm.de; 5 Department of Neurosurgery, Ulm University Medical Center, 89081 Ulm, Germany; georg.karpel@gmail.com

**Keywords:** glioblastoma, gamitrinib, HDAC inhibitors, tumor metabolism, electron transport chain, Bcl-2 family

## Abstract

The heterogeneity of glioblastomas, the most common primary malignant brain tumor, remains a significant challenge for the treatment of these devastating tumors. Therefore, novel combination treatments are warranted. Here, we showed that the combined inhibition of TRAP1 by gamitrinib and histone deacetylases (HDAC1/HDAC2) through romidepsin or panobinostat caused synergistic growth reduction of established and patient-derived xenograft (PDX) glioblastoma cells. This was accompanied by enhanced cell death with features of apoptosis and activation of caspases. The combination treatment modulated the levels of pro- and anti-apoptotic Bcl-2 family members, including BIM and Noxa, Mcl-1, Bcl-2 and Bcl-xL. Silencing of Noxa, BAK and BAX attenuated the effects of the combination treatment. At the metabolic level, the combination treatment led to an enhanced reduction of oxygen consumption rate and elicited an unfolded stress response. Finally, we tested whether the combination treatment of gamitrinib and panobinostat exerted therapeutic efficacy in PDX models of glioblastoma (GBM) in mice. While single treatments led to mild to moderate reduction in tumor growth, the combination treatment suppressed tumor growth significantly stronger than single treatments without induction of toxicity. Taken together, we have provided evidence that simultaneous targeting of TRAP1 and HDAC1/2 is efficacious to reduce tumor growth in model systems of glioblastoma.

## 1. Introduction

Patients suffering from glioblastoma (GBM), the most common primary malignant brain tumor, still require more efficient therapies to extend the so far unsatisfying overall survival between twelve to fifteen months [1,2]. Although significant progress has been made with regards to the molecular characterization, this knowledge, thus far, has not led to fruition in terms of better treatments. What remains clear is that glioblastomas are amongst the most heterogeneous cancers and this pivotal observation clearly suggests that drug combination treatments are likely the key to achieve a better prognosis for this disease [1,2,3].

Proceeding with this premise, we have conducted a high-throughput drug screen and found that mitochondrial matrix chaperone inhibitors that are in preparation for clinical testing cause synthetic lethality in the presence of selective or global histone deacetylase (HDAC) inhibitors in cancer cells [4]. Represented by the lead compound gamitrinib (GTPP) mitochondrial matrix chaperone inhibitors have shown anti-cancer activity in several tumor model systems, including prostate cancer, colon cancer, lymphoma, leukemia and glioblastoma [5,6,7]. With regards to the underlying mechanisms, gamitrinib was linked to mitochondrial permeability related cell death akin to its related predecessor, the Hsp90 inhibitor peptide shepherdin [8,9], which interferes with both cytosolic and mitochondrial Hsp90. Another compelling mechanism is the notion that TRAP1 (the molecular target of gamitrinib) is a major chaperone for the respiratory complexes of the electron transport chain and consequently interference with TRAP1 disintegrates oxidative phosphorylation [10]. Global (panobinostat) and selective (romidepsin (HDAC1 and 2)) HDAC inhibitors are FDA-approved drugs, known for their efficacy against multiple myeloma [11]. However, to date, these molecules have fallen short of expectation in the context of glioblastoma treatment [12], emphasizing the need for combination treatments. Recent findings from our lab have highlighted that in GBM model systems HDAC inhibitors block glycolysis and in turn activate oxidative phosphorylation fueled by fatty acid oxidation to counteract cell death and inhibition of proliferation [13]. Therefore, blocking the electron transport chain by TRAP1 antagonist may likely enhance the killing effect of FDA approved HDAC inhibitors in GBM cells.

Here, we show that pan and selective HDAC inhibition is indeed synthetically lethal with TRAP1 inhibition in various model systems of glioblastoma, including patient-derived xenograft (PDX) cells. Mechanistically, this occurs through several processes, including the induction of metabolic stress by interference with tumor cell energy metabolism accompanied by modulation of pro- and anti-apoptotic Bcl-2 family proteins and the induction of a cell death with apoptotic features.

## 2. Material and Methods

### 2.1. Cell Cultures and Growth Conditions

The indicated cell cultures were grown and maintained at 37 °C supplemented with 5% CO_2_. U87, LN229 and T98G cell lines were obtained from the American Type Culture Collection (Manassas, VA, USA). U251 cell line was obtained from Sigma (St. Louis, MO, USA). GBM12, GBM14 and GBM43 were obtained from Dr. Jann Sarkaria (Mayo Clinic, Rochester, MN, USA). Cells were cultured in Dulbecco’s Modified Eagle Medium (DMEM) (Fisher Scientific (Waltham, MA, USA), MT10013CV), supplemented with 10% FBS (Gemini, FBS002) and 100 μg/mL of Primocin (Invivogen (San Diego, CA, USA), ant-pm-1). For the treatment experiment, cells were cultured in DMEM containing 1.5% FBS and primocin. The U87 PbR were exposed with 100 nM panobinostat for a week.

### 2.2. Reagents

The TRAP1 inhibitor, Gamitrinib (GTPP), was a gift from Dr. Dario Altieri (Wistar Institute, Philadelphia, PA, USA). Z-VAD-FMK (HY-16658B), panobinostat (HY-10224) and romidepsin (HY-15149) were purchased from MedChemExpress (Monmouth Junction, NJ, USA). A 10 mM working solution in dimethylsulfoxide (DMSO) was prepared for all reagents prior to storage at −20 °C. Final concentrations of DMSO were below 0.1% (*v*/*v*).

### 2.3. Cell Viability Assays

Cells were seeded in 96-well plates and allowed to attach overnight. Cells were treated with different doses of indicated drugs for 72 h and CellTiter-Glo^®^ assays (Promega (Madison, WI, USA), G7571) were performed according to the manufacturer’s instruction. We employed the median-effect equation (Chou-Talalay), to evaluate drug synergy. Following calculations, this approach yields normalized isobolograms and the combination index (CI), respectively [14].

### 2.4. Flow Cytometry

Cells were treated with drug compounds for the indicated time frames and processed for staining in accordance with the manufacturers’ instructions. To detect apoptosis, the Annexin V Apoptosis Detection Kit (BD Pharmingen (Franklin Lakes, NJ, USA), BD 556419) was used. To measure the subG1/G0 fraction following treatments, the Propidium Iodide (PI)/RNase Staining Solution was employed (Cell Signaling Technology (Danvers, MA, USA), CST 4087S). To detect mitochondrial membrane potential, Mitochondrial Membrane Potential kit (CST 13296S) was used. The FlowJo software (version 10.6.2; Tree Star, Ashland, OR, USA) was employed for the entire analysis of the collected data.

### 2.5. Transfection of siRNAs

Transfections were performed with Lipofectamine RNAiMAX (Invitrogen (Carlsbad, CA, USA), 13778075) according to the manufacturers’ instructions. Non-targeting siRNA-pool (D-001810-10-20), siTRAP1 (L-010104-00-0005), siBax (L-003308-01-0005) and siATF4 (L-005125-00-0005) were purchased from Dharmacon (Lafayette, CO, USA). siPMAIP1 (siNoxa, 4392420) and siBAK (4390824) were purchased from Ambion (ThermoFisher, Waltham, MA, USA).

### 2.6. Extracellular Flux Analysis

Both the oxygen consumption rate (OCR) and the extracellular acidification rate (ECAR) were determined on the Seahorse XFe24 analyzer in accordance with the manufacturers’ instructions (Agilent Technologies, Santa Clara, CA, USA). XFe24 cell culture microplates (Agilent) were used for the experiments and U87 GBM cells were cultured in DMEM containing 5 mM Glucose, 1 mM Glutamine and 10% FBS. Following adherence overnight, treatments (24 h) were administered in the same medium except that 1.5% FBS was used. Mitochondrial stress assay was performed in the Seahorse XF base medium (Agilent, 102353-100) containing 10 mM glucose, 2 mM glutamine, 1 mM pyruvate. The following compounds were injected in a sequential order—2 μmol/L oligomycin (OM), 2 μmol/L Carbonyl cyanide-4 (trifluoromethoxy) phenylhydrazone (FCCP) and 0.5 μmol/L rotenone/antimycin (R/A).

### 2.7. Western Blot Analysis

Protein expression was determined by western blot analysis (Invitrogen, NP0321BOX) or protein capillary electrophoresis (Protein Simple (San Jose, CA, USA) SM-W004). Primary antibody incubations were performed overnight at 4 °C. For standard western blot, the following antibodies were applied PARP (CST 9532; 1:500); cCP9 (CST 7237; 1:500); cCP3 (CST 9665; 1:500); Tubulin (Sigma T5168; 1:1000); Mcl-1 (CST 5453; 1:500); Bcl-xL (CST 2764; 1:500); Bcl-2 (CST 4223; 1:500); BIM (CST 2933; 1:500), Noxa (Calbiochem (Burlington, MA, USA) OP180, clone 114C307; 1:500), Vinculin (Abcam (Cambridge, MA, USA) ab129002, 1:500), OXPHOS (Abcam ab110411;1:500); β-actin (Sigma Aldrich A1978, clone AC15; 1:2,000). TRAP1 (Novus Biological (Littleton, CO, USA), NBP2-20700, 1:500), SDHA (Abcam ab123545; 1:500), SDHB (Abcam ab154974; 1:500). For protein capillary electrophoresis, the following antibodies were applied ATF3 (Novus Biological NBP1-85816; 1:25); ATF4 (CST 11815; 1:25), GRP78 (BiP-CST 3177; 1:100); elF2α (CST 9722; 1:25), p- elF2α (CST 3398; 1:25), CHOP (CST 2895; 1:25), Noxa (Calbiochem OP180, clone 114C307; 1:25). The HRP linked secondary antibodies were from Santa Cruz Biotechnology Inc. Western blots were visualized on the Azure (C300) imaging system (chemiluminescence based). The uncropped images are quantified and presented in the Supplementary section.

### 2.8. Real-Time PCR Analysis

The miRNAeasy Mini Kit (QIAGEN 217004) was employed to harvest RNA following treatments. Following capture of the RNA, reverse-transcription was performed (cDNA synthesis kit (Quantabio 101414-106)). The SYBR green Real time PCR (RT-PCR) reagents kit (Quantabio 101414-276) was utilized as the reaction mix. The settings for the RT-PCR machine (Quantabio) are as follows—95 °C for 10 min, followed by 40 cycles of 95 °C for 15 s, 60 °C for 30 s and 72 °C for 30 s. The fold changes were calculated based on 18S in the threshold cycle (Cq). Pertinent primer sequences are indicated in Table 1.

### 2.9. Chromatin Immunoprecipitation (CHIP) Sequencing

Chromatin immunoprecipitation (CHIP) sequencing was performed following the company’s instructions (SimpleChIP^®^ Enzymatic Chromatin IP Ki, CST 9003). Chromatin was immune-precipitated with the following antibodies—H3K27ac (CST 4535, 10 μL/sample), H3K27Me3 (CST 9733, 10 μL/sample), Rpb1 (CST 14958, 10 μL/sample) or Rabbit IgG (CST 2729, 2 μL/sample). Following purification, the ChIP DNA was assessed for quality by real time PCR analysis. Libraries were prepared and were subjected to next generation sequencing (Illumina HiSeQ instrument; HiSeq 4000; single read 50 bp (SR50)). Mapping to the human genome (hg38) was performed by bowtie2. Peaks were called and obtained with MACS2 software. The BEDgraph files were converted to the bigwig format and visualized with the Integrated Genome Browser (IGB). The experiment is deposited (GSE124877 and GSE150395).

### 2.10. Subcutaneous Xenograft Model

GBM12 and GBM43 glioblastoma patient-derived xenograft (PDX) tumors were injected into the flanks of 6–8 weeks old Nu/Nu mice. Several subcutaneous tumors on each mouse were implanted. Tumor and weight measurements were performed three times a week followed by intraperitoneal administration of the compounds. The following dosages were administered—Panobinostat: 5 mg/kg; Gamitrinib: 3 mg/kg. Drugs were dissolved in a mixture of drug (dissolved in DMSO), Kolliphor EL (Sigma, 61791-12-6), Ethyl Alcohol 200 Proof (Pharmco-Aaper, 64-17-5) and PBS at the ratio: 10:32:8:50 (*v*/*v*/*v*/*v*). The length and width were obtained with a caliper and subsequent calculation based on the formula—(length x width^2^)/2. Weight measurements were only performed on three mice per group.

### 2.11. TUNEL and Ki67 Staining

The specimens were fixed in formalin and embedded in paraffin. Following dewaxing and rehydration, the tumors were exposed to proteinase K (Agilent Dako, S3020). The processed slides were exposed to TUNEL reaction mixture and the reaction was terminated in the converter peroxidase (POD) solution. The chromogen, diaminobenzidine, was employed for visualization of the TUNEL reaction. Background nuclear staining was established with hematoxylin. For Ki67 staining, samples were incubated with Ki67 antibody (Agilent Dako, GA626).

### 2.12. Statistical Analysis

Statistical significance was assessed by Student’s *t*-test or ANOVA (for multiple comparison) using Prism 8 (GraphPad, La Jolla, CA, USA). A *p* ≤ 0.05 was set as the level of statistical significance. * *p* < 0.05, ** *p* < 0.01, ***/**** *p* < 0.001 while n. s. means not significant.

### 2.13. Study Approval

All procedures were in accordance with Animal Welfare Regulations and approved by the Institutional Animal Care and Use Committee at the Columbia University Medical Center (AC-AABC6505).

## 3. Results

### 3.1. FDA Approved HDAC Inhibitors and the Mitochondrial Chaperone Inhibitor, Gamitrinib, Lead to a Synergistic Reduction of Cellular Viability in Glioblastoma Models

**Figure 1:**
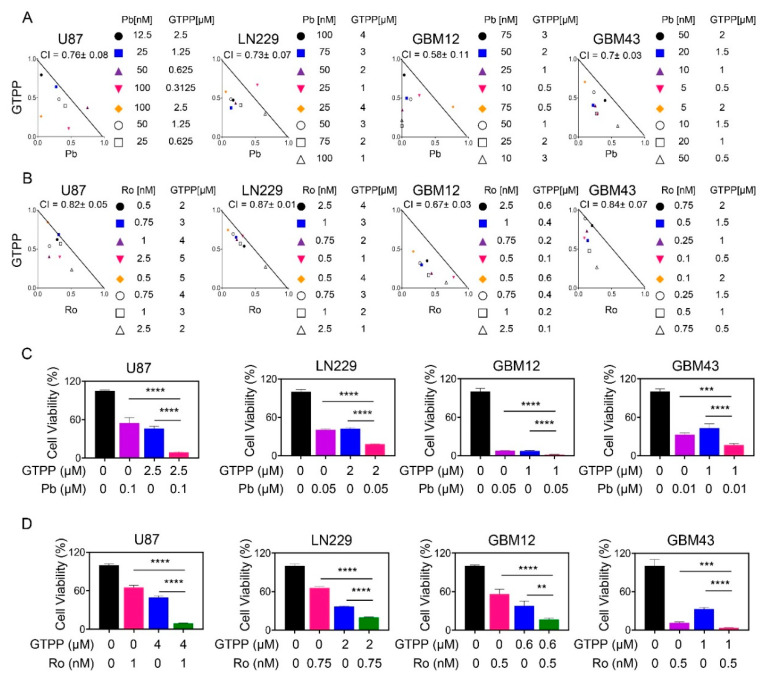
Combined treatment with gamitrinib and histone deacetylase (HDAC) inhibitors elicits synergistic reduction in cellular proliferation of glioblastoma (GBM) cells. (**A**,**B**) U87, LN229, GBM12 and GBM43 cells were treated with gamitrinib (GTPP), panobinostat (Pb)/romidepsin (Ro) or the combination of GTPP and panobinostat/romidepsin for 72h. Thereafter, cellular viability and statistical analysis were performed. Isobolograms are shown; (**C**,**D**) The graphs show cellular viability data following treatment with vehicle, panobinostat/romidepsin, gamitrinib or the combination for 72h in the indicated GBM cells (*n* = 3, 4). Shown are means and SD. ANOVA was used for statistical analysis. ** *p* < 0.01, ***/**** *p* < 0.001.

Informed by a drug screen approach to define synthetic lethal interaction for the novel TRAP1 inhibitor, gamitrinib, we validated whether or not global or selective HDAC inhibitors induce synergistic reduction of cellular viability in relevant model systems of human glioblastoma (Figure 1A–D). To this purpose, we assessed cellular viability following treatment with the global HDAC inhibitor panobinostat, gamitrinib (GTPP) and the combination of both reagents. While single treatment impacted the survival, the combination treatment led to a synergistic reduction of cellular viability in established glioblastoma cells, U87 and LN229 (Figure 1A,C). This occurred in a similar fashion, suggesting that the genetic make-up of these tumor cells likely does not contribute to the efficacy of the combination treatment in light of the fact that U87 are wild type *TP53*, whereas in contrast LN229 are mutated. The TP53 status is relevant in that GBMs are commonly mutated in TP53, which may impact response and resistance to therapy [1,2,3]. Similar observations were made in T98G that harbors *TP53* mutations (Figure S1A,B). We extended our experiments to a more clinically relevant scenario [15] by employing short term patient-derived xenograft cell cultures, GBM12 and GBM43 (Figure 1A,C). Compared to the established cell cultures, the GBM12 cells revealed a relatively pronounced susceptibility to both gamitrinib and panobinostat. Nevertheless, the combination treatment still resulted in a synergistic growth reduction. Following treatment with gamitrinib and panobinostat, the GBM43 cell cultures revealed a synergistic loss of cellular viability as well. These results suggest that the combination treatment of global HDAC inhibitors in combination with TRAP1 inhibitors are effective in reducing the viability of a variety of GBM cells, likely to be irrespective of *TP53* status.

A certain concern in drug combination therapies relates to off target effects, which in part is implied by the term “global” HDAC inhibitors. Over the recent years, strategies have unfolded to block targets in a more precise manner. Within the group of HDAC inhibitors, the FDA approved compound, romidepsin, comes closer to this paradigm given that it inhibits both HDAC1 and HDAC2 in the low nanomolar range. Consistently, we applied these low nanomolar concentrations of romidepsin for our drug combination studies with gamitrinib. In the context of established GBM culture systems, romidespin displayed a remarkable efficacy to reduce the cellular viability, which occurred in the very low nano molar range. Remarkably, when romidepsin was combined with gamitrinib the reduction was further enhanced in a synergistic manner in both *TP53* wild type U87 as well as mutated LN229 and U251 GBM cells, respectively (Figure 1B,D and Figure S1C,D). Akin to panobinostat, we evaluated the efficacy of single and combination treatments, involving romidepsin and gamtrinib, in short term patient-derived xenograft cultures (Figure 1B,D and Figure S1C,D). In alignment with the results obtained in established GBM cells romidepsin exerted a remarkable reduction of cellular viability (again in the low nano molar range), which was synergistically enhanced by gamitrinib in both GBM12, GBM43 and GBM14 cells.

### 3.2. Dual Inhibition of TRAP1 and HDAC Elicits Enhanced Activation of a Cell Death with Apoptotic Features

**Figure 2:**
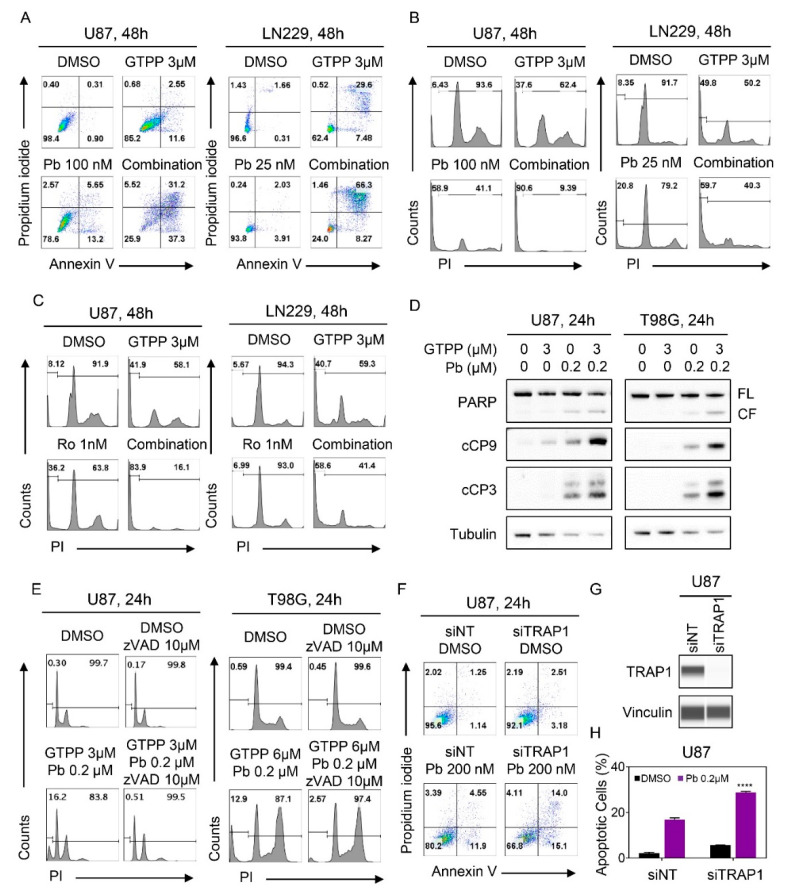
Combined inhibition of TRAP1 and HDACs enhanced activation of a cell death with apoptotic features, including cleavage of caspases. (**A**) U87 and LN229 cells were treated with the indicated concentration of gamitrinib, panobinostat or the combination of both for 48h. Thereafter, cells were labeled with annexin/propidium iodide (PI) dye and analyzed by multi-parametric flow cytometry. Shown are representative flow plots; (**B**,**C**) U87 and LN229 cells were treated with the indicated concentrations of gamitrinib, panobinostat/romidepsin or the combination of both for 48h. Thereafter, cells were labeled with propidium iodide (PI) dye and analyzed by flow cytometry. Shown are representative flow plots; (**D**) Standard western blots of cell lysates of U87 and T98G treated with gamitrinib, panobinostat or the combination of both for 24 h. Tubulin is used as a loading control. FL: full length, CF: cleaved fragment; (**E**) U87 and T98G cells were treated with the combination treatment of gamitrinib and panobinostat in the presence or absence of zVAD for 24h. Thereafter, cells were labeled with propidium iodide (PI) dye and analyzed by flow cytometry. Shown are representative flow plots; (**F**–**H**) U87 GBM cells were transfected with scrambled or TRAP1 specific siRNA and treated with panobinostat for 24h. Knockdown efficiency was confirmed by protein capillary electrophoresis. Vinculin serves as a loading control. Thereafter, cells were labeled with Annexin/PI dye and analyzed by multi-parametric flow cytometry (*n* = 3). Shown are means and SD. Statistical significance was determined by two-tailed Student’s *t*-test. **** *p* < 0.001.

While single treatment with gamitrinib and panobinostat elicited cell death, the combination treatment was significantly more potent in established and PDX GBM cells as shown by two different flow cytometric based cell death assays (Figure 2A,B, Figure S2A,B and Figure S3A–C). To confirm that selective HDAC1/2 inhibition phenocopies the effects seen with the global HDAC inhibitor we tested the combination treatment of gamitrinib and romidepsin in established and patient derived GBM cells as well (Figure 2C and Figure S3C–F). In keeping with the earlier observations, combined treatment with gamitrinib and romidepsin exerted significantly more DNA fragmentation (apoptotic cell death) than vehicle and single treatments (Figure 2C and Figure S3C–F). Apoptotic cell death is usually preceded by loss of mitochondrial membrane potential that is associated with activation of the intrinsic apoptotic cascade. Concordant to the apoptosis related results, we observed enhanced loss of mitochondrial membrane potential following combined treatment with gamitrinib and panobinostat across all the cell cultures tested (Figure S4A,B).

Finally, we asked whether the combination treatment would display enhanced cleavage/activation of caspases and caspase substrate cleavage, respectively. While panobinostat revealed some cleavage of initiator caspase-9, effector caspase 3 and PARP (substrate of activated caspase 3), the combination treatment enhanced this further in U87, T98G and U251 GBM cells, in keeping with the results of the flow cytometry based cell death assays (Figure 2D and Figure S4C,D). One of the issues in cell death related research is to determine the requirements of the observed form of cell death, for example, whether for instance the activation of caspases is indeed involved in the process or merely a bystander effect. To address this question, we utilized the pan-caspase inhibitor, zVAD-fmk. We found that DNA fragmentation induced by the combination treatment of gamitrinib and panobinostat was rescued by concomitant treatment with zVAD-fmk, suggesting that caspases are indeed involved and necessary in the process (Figure 2E and Figure S4E).

Although gamitrinib has been coined as a TRAP1 inhibitor we sought to confirm whether selective genetic inhibition is sufficient to enhance HDAC inhibitor mediated induction of cell death. To this purpose, we transfected U87 GBM cells with non-targeting or TRAP1 specific siRNA. Following transfection, the cells were subjected to panobinostat treatment and following the incubation period analyzed for cell death induction by annexin V/PI based flow cytometry (Figure 2F–H). In keeping with earlier experiences, knockdown of TRAP1 on its own had very little effects on cell death induction in the U87 GBM cells. In contrast, TRAP1 silencing significantly enhanced panobinostat mediated cell death as compared to cells transfected with the non-targeting siRNA pool. In a parallel approach, we analyzed the same experimental setup for loss of mitochondrial membrane potential (TMRE staining with flow cytometric analysis) (Figure S4F,G). In keeping with the cell death findings, TRAP1 silencing in the presence of panobinostat led to enhanced dissipation of the mitochondrial membrane potential. Overall, these observations suggest that TRAP1 inhibition is sufficient to sensitize GBM cells for cell death induction by HDAC inhibitors.

### 3.3. The Combination Treatment Modulates the Expression of the Bcl-2 Protein Family Members and Pro-Apopotic Members of the Bcl-2 Family Members are Required for Cell Death Execution

**Figure 3:**
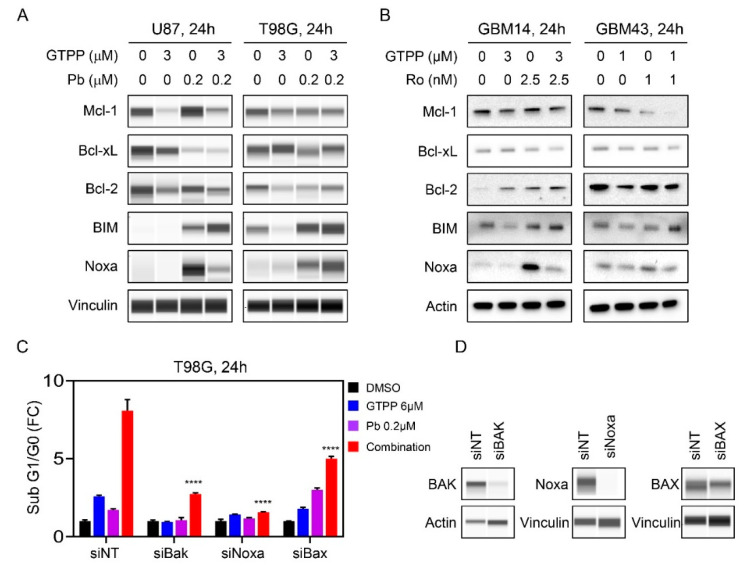
Rescue experiments demonstrate involvement of the Bcl-2 family members in cell death elicited by the combination treatment of gamitrinib and HDAC inhibitors. (**A**) U87 and T98G cells were treated with gamitrinib, panobinostat or the combination of both for 24h. Whole cell lysates were subjected for protein capillary electrophoresis. Vinculin serves as a loading control; (**B**) GBM14 and GBM43 cells were treated with gamitrinib, romidepsin or the combination of both for 24h. Whole cell lysates were subjected to standard western blotting. Actin is a loading control; (**C**) T98G cells were transfected with non-targeting (NT) siRNA, BAK, Noxa or BAX specific siRNAs. Following transfection, the cells were exposed to vehicle, gamitrinib (GTPP), panobinostat or the combination treatment of gamitrinib and panobinostat for 24h. Thereafter, cells were labeled with propidium iodide (PI) dye and analyzed by flow cytometry (*n* = 3). Shown are means and SD. (**D**) Shown are protein capillary electrophoresis analyses of T98G cells transfected with the indicated siRNAs. ANOVA was used for statistical analysis. **** *p* < 0.001.

Intrinsic apoptosis is modulated in part by Bcl-2 family members since they determine the permeability of the outer mitochondrial membrane. Given that we noted a cell death with features of apoptosis induced by the combination treatment, we evaluated the expression of the anti-apoptotic Bcl-2 family members, Bcl-2, Bcl-xL and Mcl-1 in established and PDX GBM cells by utilizing protein capillary electrophoresis and standard western blotting. In most instances, we noted a suppression of the three anti-apoptotic Bcl-2 family members in established GBM cells (U87, T98G and U251) following treatment with the combination treatment of gamitrinib and panobinostat as compared to vehicle treatment (Figure 3A and Figure S5A–D). In contrast, pro-apoptotic Bcl-2 family members remained either unchanged or increased. Next, we assessed how the combination treatment of gamitrinib and the selective HDAC inhibitor, romidepsin, affects the expression of the Bcl-2-family members of proteins. To this end, we treated the established and PDX GBM cells with the gamitrinib, romidepsin or the combination of both (Figure 3B and Figure S5E,F). Akin to panobinostat, we found that the combination treatment led to a decline in most anti-apoptotic proteins. We noted an exception to this general pattern in GBM14 cells, in which there was an increase in Bcl-2 following the combination treatment (Figure 3B and Figure S5E,F). With regards to the pro-apoptotic Bcl-2 family members, we noted an up-regulation of BIM. Concerning Noxa, the expression pattern was more complicated with some showing increased or constant expression or decreased levels (Figure 3B and Figure S5E,F). When considering the ratio between pro-apoptotic Noxa and anti-apoptotic Mcl-1, the combination treatment resulted in an increased ratio between the proteins in most instances when compared to the vehicle treatments but for the most part no differential engagement between combination treatments and single treatments (Figure S5A,C,F).

Next, we assessed whether the pro-apoptotic Bcl-2 family members are required for the induction of cell death in the context of the combination treatment. To this purpose, we initially focused on pro-apoptotic BAX and BAK proteins since these molecules are the mediators to permeabilize the outer mitochondrial membrane. T98G cells were transfected with non-targeting or specific siRNA against BAX and BAK. Thereafter, cells were treated with vehicle, gamitrinib, panobinostat or the combination treatment of gamitrinib and panobinostat (Figure 3C,D). While the single treatments elicited induction of cell death, the response was substantially further enhanced when gamtrinib and panobinostat were administered together (Figure 3C). It is notable that the single treatments were rescued by BAK silencing but only minimal or not at all by BAX silencing (Figure 3C). Whereas we detected a significant amount of cell death induction by the combination treatment in the non-targeting condition, reduced cell death was measured when either BAX or BAK were silenced. As we noted regulation of Noxa by the combination treatment we silenced Noxa by siRNA as well. Following transfection, Noxa silencing protected from gamtrinib/panobinostat enhanced cell death (Figure 3C,D). These results support the notion that Bcl-2 family members are involved in the cell death induced by the combination treatment of gamitrinib and HDAC inhibitors. However, we acknowledge that despite the involvement of the Bcl-2 family members and caspases in the cell death elicited by the combination treatment we did not detect a bona-fide differential engagement of the Bcl-2 family members in the combination treatment as compared to the single treatments.

### 3.4. The Combination Treatment of Gamitrinib and HDAC Inhibitor Mediates an Integrated Stress Response

**Figure 4:**
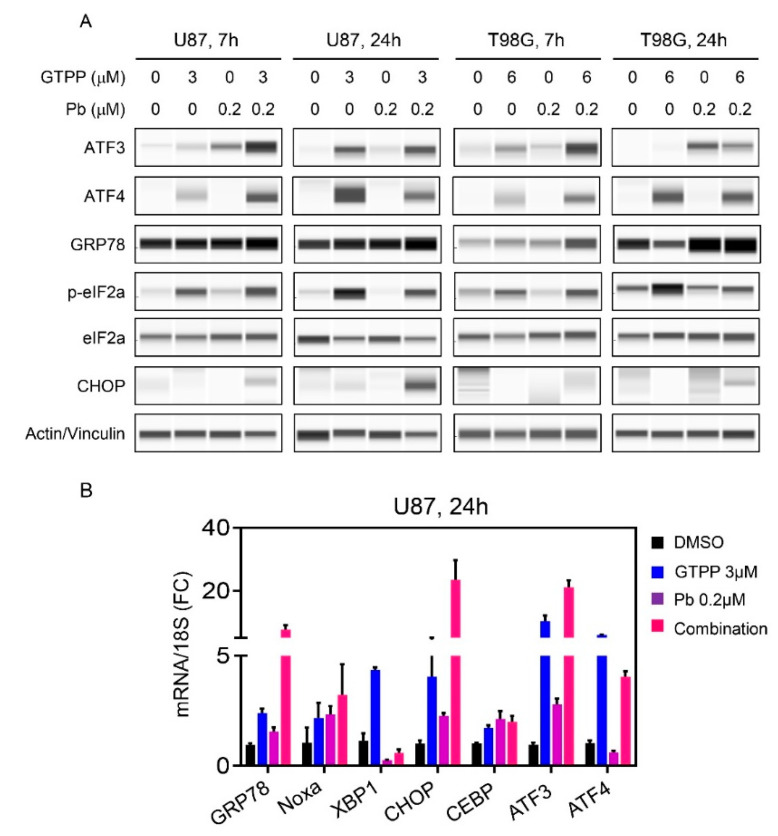
The combination treatment of gamitrinib and HDAC inhibitors initiates an integrated stress response that partially facilitates Noxa expression. (**A**) U87 and T98G cells were treated with gamitrinib, panobinostat or the combination for 7h and 24h. The whole cell lysates were subjected to protein capillary electrophoresis. Actin/Vinculin serve as loading controls where indicated; (**B**) Real time PCR analysis of stress response related mRNAs in U87 GBM cells treated with gamitrinib, panobinostat or the combination of both for 24h (*n* = 3). Shown are means and SD.

The induction of cell death and the modulation of Bcl-2 family members is often preceded and regulated by the so called integrated stress response, which may be induced by a number of factors, including loss of energy and the accumulation of misfolded proteins. To this end, we treated established U87 and T98G GBM cells with vehicle, gamitrinib, panobinostat or the combination of both for either 7 h or 24 h (Figure 4A). Thereafter, proteins were harvested and subjected to capillary electrophoresis. We noted an early increase in GRP78 protein levels accompanied by enhanced phosphorylation of eif2α predominantly evident in the combination treatment at 7 h (Figure 4A). Concomitantly, we also identified an increase in ATF4 and ATF3, respectively, which is in support with the general accepted notion that eif2α is upstream of ATF4 and when eif2α is phosphorylated it favors ATF4 translation and certain other stress related proteins. Downstream of ATF4 lies CHOP and we detected an increase in CHOP levels, especially in U87 cells (Figure 4A). It is noteworthy that the integrated stress response signaling was activated much faster by the combination treatment and at 24 h the difference between combination treatment and single treatment was not as prominent anymore, indicating this process is an early event (Figure 4A). We further validated on the transcriptional levels the activation of the integrated stress response as well (Figure 4B). To this purpose, U87 GBM cells were treated with vehicle, gamitrinib, panobinostat or the combination of both for 24 h. Following treatment, RNA was isolated and transcript levels for several factors related to the integrated stress response, including XBP1 and CEBPB (two additional down-stream modulators of the cellular stress response), were analyzed. Similarly, to the protein expression analysis, we noted mRNA increases of stress response related transcripts. From this analysis, it emerged that both ATF4 and ATF3 are up-regulated at the transcript level as well, suggesting that the resulting protein increase is not only related to enhanced and preferential translation but also linked to up-regulated transcription. This also included an increase in Noxa accompanied by an up-regulation of ATF4, potentially highlighting a transcriptional modulation of Noxa by ATF4 [16,17] (Figure 4B).

### 3.5. Combined Inhibition of HDAC and TRAP1 Modulates Tumor Cell Metabolism

**Figure 5:**
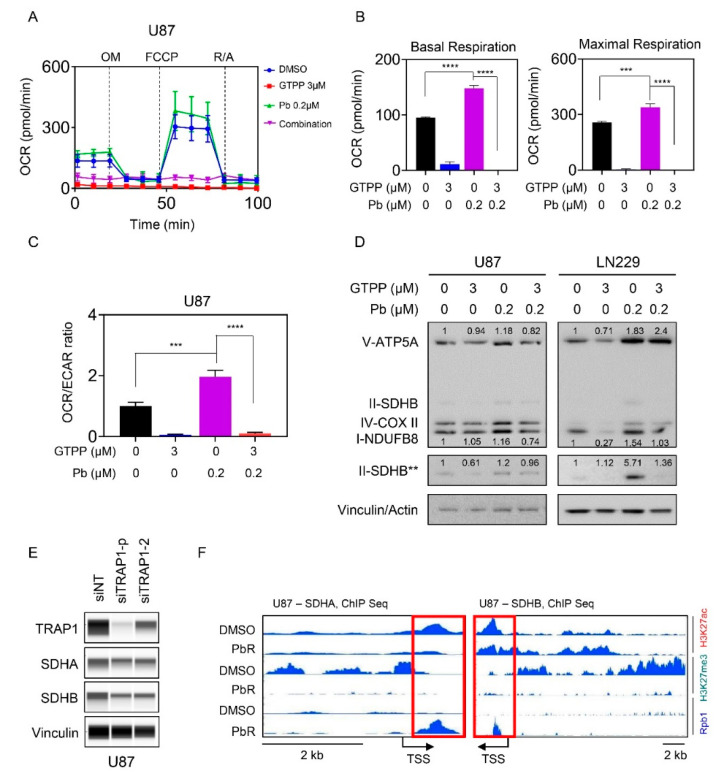
The combination treatment of gamitrinib and HDAC inhibitors interferes with GBM cell energy metabolism. (**A**) Mitochondrial stress test of U87 cells treated with gamitrinib, panobinostat or the combination of both for 24 h. OM: oligomycin, FCCP: Carbonyl cyanide-4 (trifluoromethoxy) phenylhydrazone, R/A: rotenone and antimycin A (*n* = 4); (**B**) The basal respiration and maximal respiration from experiment in (A) were calculated (*n* = 3, 4); (**C**) Shown is the OCR/ECAR ratio from mitochondrial stress test of U87 cells treated with the indicated concentrations of gamitrinib, panobinostat or the combination of both for 24 h (*n* = 4). OCR: oxygen consumption rate, ECAR: extracellular acidification rate (a surrogate for glycolytic activity); (**D**) U87 and LN229 cells were treated with gamitrinib, panobinostat or the combination of both for 24 h and the whole cell lysates were subjected to protein capillary electrophoresis. The double asterisk indicates a stronger exposure of SDHB. Vinculin is the loading control for U87 and actin is the loading control for LN229. The expression levels of V-ATP5A, I-NDUFB8 and II-SDHB were quantified by using ImageJ (shown in cursive font); (**E**) U87 cells were transfected with non-targeting (siNT) or TRAP1 specific siRNA (siTRAP1)(single or pool (p)) and the whole cell lysates were subjected to protein capillary electrophoresis; (**F**) Shown are the respective tracks of ChIP sequencing around SDHA and SDHB of parental and panobinostat treated U87 GBM cells. Highlighted is the transcriptional start site (TSS) by a red rectangle. Shown are means and SD. ANOVA was used for the statistical analysis. ***/**** *p* < 0.001.

Based on the literature and our own work, the integrated stress response is known to be related to metabolism and our earlier work demonstrated a role for gamitrinib as well as panobinostat in GBM metabolism [13,18]. Given that these effects are contrary to tumor cell respiration (i.e., panobinostat increases, whereas gamitrinib suppresses the oxygen consumption rate (OCR)) through disruption of the complex II of the respiratory chain (SDH), we hypothesized that gamitrinib might suppress panobinostat mediated increase in oxygen consumption rate. To address this question, we utilized the mitochondrial stress assay on the seahorse analyzer in U87 GBM cells. In this assay, basal OCR measurements are taken, followed by injection of oligomycin (ATP-production), FCCP (maximal respiration) and rotenone/antimycin (inhibition of mitochondrial respiration). After each injection of these compounds the OCR values are recorded. U87 GBM cells were treated with vehicle, gamitrinib, panobinostat or the combination of both (Figure 5A–C). Following treatment, the cells were subjected to extracellular flux analysis. Basal measurements, maximal respiration and the OCR/ECAR (extracellular acidification rate) ratio revealed an increase following treatment with panobinostat and a decrease with gamitrinib (Figure 5B,C). The increase of the OCR/ECAR ratio by panobinostat is in keeping with the reversal of the Warburg-effect, leading to suppression of ECAR and an increase of OCR. Importantly, gamitrinib reversed panobinostat mediated increase of basal OCR, maximal respiration and the OCR/ECAR ratio (Figure 5B,C). These data suggest that the combination treatment reduced energy metabolism in GBM cells. Next, we asked about the mechanism by which panobinostat facilitates an increase in OCR. While there are several possibilities, the first and foremost to consider are the expression levels of respiratory complexes. To this end, U87 and LN229 GBM cells were treated with vehicle, gamitrinib or the combination of both and following protein isolation analyzed for the expression levels of the five respiratory complexes (Figure 5D and Figure S6A–C). While we detected an increase of respiratory complexes following panobinostat treatment, gamitrinib counteracted this up-regulation, which is in agreement with our findings obtained on the seahorse analyzer (Figure 5D and Figure S6A–C). To validate that TRAP1 is directly involved in this phenomenon mediated by gamitrinib, we silenced TRAP1 in U87 cells by two specific siRNAs (Figure 5E). Following transfection, protein expression of both SDHA and SDHB was assessed by protein capillary electrophoresis, demonstrating that TRAP1 silencing sufficed to suppress these two proteins that are associated with complex two of the respiratory chain (Figure 5E). Previous findings from our group demonstrated that panobinostat up-regulated SDHA and SDHB following acute and chronic treatment with panobinostat and that the up-regulation is mediated on a transcriptional level [13]. Given that HDAC inhibitors affect the post-translational status of histones and histones regulate the accessibility of the chromatin we assessed the status of H3K27ac, H3K27me3 and RNA polymerase II (Rpb1) binding to the SDHA and SDHB locus by chromatin immunoprecipitation coupled with next generation sequencing of vehicle or panobinostat treated cells (Figure 5F). Regarding the SDHB locus, we noticed a modulation of H3K27ac and a striking reduction of the repressive histone mark, H3K27me3 in panobinostat exposed U87 cells (PbR). Consistently, this phenomenon was coupled with enhanced presence of Rbp1 at the transcriptional start site of SDHB, facilitating mRNA production of SDHB (Figure 5F). Similar findings were observed at the SDHA locus.

### 3.6. The Combination Treatment of Gamitrinib and Panobinostat Reduces Tumor Growth More Potently than Single Treatments in Glioblastoma PDX Models in Mice

**Figure 6:**
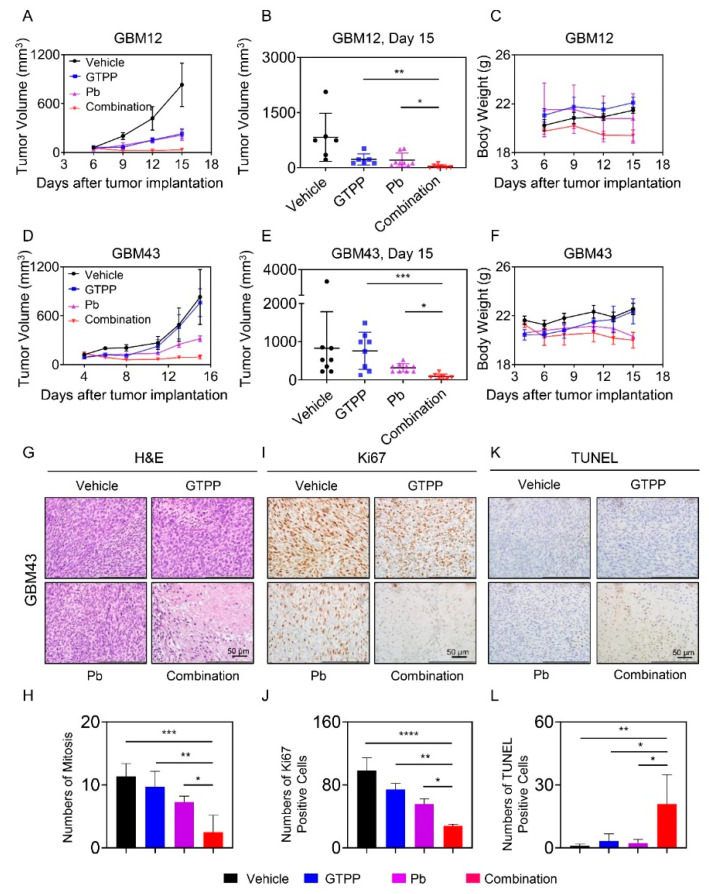
Combined treatment with gamitrinib (GTPP) and HDAC inhibitor, panobinostat, elicits enhanced tumor growth inhibition in vivo. (**A**) Patient-derived xenograft tumors, GBM12 cells were implanted into the subcutis of immunocompromised Nu/Nu mice. After the tumors were established, randomization was performed to define four treatment groups: vehicle, gamitrinib (3 mg/kg), panobinostat (5 mg/kg) and the combination treatment. Shown are the tumor volumes over time; (**B**,**C**) The graphs show tumor volume (*n* = 6–8) on the last day and the body weight (*n* = 3) of the experiment in (A); (**D**) Patient-derived xenograft tumors, GBM43 cells were implanted into the subcutis of immunocompromised Nu/Nu mice. After the tumors were established, randomization was performed to obtain four treatment groups: vehicle, gamitrinib (3 mg/kg), panobinostat (5 mg/kg) and the combination treatment. Shown are the tumor volumes over time; (**E**,**F**) The graphs show tumor volume (*n* = 7–9) on the last day and the body weight (*n* = 3) of the experiment in (**D**); (**G**–**L**) Tumors from the experiment in (**D**) were stained with H&E, TUNEL or Ki67. Quantifications are provided from several high-power fields in H, J and L. Shown are means and SD. ANOVA was used for the statistical analysis. * *p* < 0.05, ** *p* < 0.01, ***/**** *p* < 0.001.

Finally, we sought to determine whether the combination treatment of gamitrinib and panobinostat could lead to the reduction of tumor growth in two patient-derived xenograft models of human GBM in mice. To this end, we implanted the GBM12 PDX line in the flank of nude mice. Following establishment of the tumors, four groups were formed, consisting of vehicle, gamitrinib (GTPP), panobinostat (Pb) or the combination of both. We noted that single treatment caused a reduction of tumor growth already (Figure 6A–C and Figure S7A). However, the combination treatment was even more effective in limiting tumor growth compared to single treatments with either gamitrinib or panobinostat (Figure 6A–C and Figure S7A). In a second GBM PDX model (GBM43), we made slightly different findings. In the GBM43 model, we found a reduction of tumor growth only mediated by panobinostat but not gamitrinib (Figure 6D–F and Figure S7B). Nevertheless, the combination treatment led to a potent further reduction of tumor growth as compared to panobinostat. These results demonstrate that the effect of the combination treatment on tumor growth is not limited to just one model, consistent with our in vitro observations. Notably, we found no significant reduction in animal weight and consistently, there was no damage imposed by the combination treatment on major organ systems, such as heart, lung, liver, kidney and pancreas as assessed by standard histological analysis (Figure S7C). We examined the tumors as well and found that there was significant distortion of the tumor architecture with reduced mitosis and emergence of necrosis in the specimens treated with the combination treatment (Figure 6G). Moreover, a lower amount of mitosis was identified (Figure 6G,H). These observations were further confirmed by Ki67 and TUNEL staining (Figure 6I–L). While vehicle and single treatments showed many large fractions of Ki67 positive nuclei, the combination revealed far less (Figure 6I,J). In contrast, TUNEL staining demonstrated a higher number of positive nuclei in the combination treatment as compared to vehicle and single treatments (Figure 6K,L). These results implicate that the combination treatment elicited its effects by reducing proliferation and induction of cell death in a largely tumor selective manner given that major organ systems did not demonstrate signs of toxicity following the treatment.

## 4. Discussion

The elucidation of more durable treatment modalities, including novel drug combination therapies, for the treatment of GBM remains one of the highest priorities [2,3,19,20,21,22,23,24,25,26]. The present drug combination, involving gamitrinib and HDAC inhibitors, appears to be effective against a broad range of different GBM model systems, suggesting potentially broad applicability, particularly with regards to heterogeneity. With the development of next generation sequencing, single cell sequencing and the huge variety of small molecule compounds an array of opportunities has emerged [1]. Notwithstanding these techniques, tumor cell metabolism has received significant attention in the field as well in light of the notion that tumor cells develop distinct features that will allow them to grow relentlessly. One of these metabolic effects is the Warburg effect [27] and our recent work demonstrated that global and selective HDAC inhibitor disrupt this phenomenon by targeting super-enhancers in genes, such as HK2, GAPDH and ENO1 [13]. Following treatment with HDAC inhibitors we noted incomplete cell killing and the surviving GBM cells displayed a reduced glycolytic activity and in order to maintain survival they facilitated tumor respiration, which in the present and earlier work we were able to link to up-regulation of respiratory complexes [13]. In the present work, we expanded this concept further by showing that apparently HDAC inhibitor treatment results in a loss of the repressive histone mark, H3K27me, around the loci of SDH. The methyltransferase EZH2 is mostly known to modulate histone methylation and is part of the PRC2 complex [28,29]. How the PRC2 complex is related to HDACs has been recently demonstrated by others, revealing an interaction between HDAC2 and the PRC2 complex [30]. Once HDAC2 is inhibited the PRC2 complex becomes hyper-acetylated and dissociates along with its ability to methylate histones [30], which in concert may result in less methylation of H3K27 and thereby facilitate the up-regulation of genes. The current concept is also in line with the recent observations that the expression of PPARGC1A, the master regulator of oxidative metabolism, is controlled by the H3K27me3 mark [31,32].

The present work originated from the observation that based on a drug screen we made the intriguing finding that HDAC inhibitors may enhance the killing efficacy of gamitrinib in tumor cells [4]. Here, we established this concept by demonstrating that global (panobinostat) and selective (romidepsin) HDAC inhibitors combined with gamitrinib synergistically reduced the viability of a broad variety of GBM model systems. Given that gamitrinib is prominently known to interfere with the electron transport chain, we hypothesized that gamitrinib and its target TRAP1 [18] could thwart HDAC inhibitor induced activation of tumor respiration. In keeping with this notion, our findings here demonstrated that gamitrinib attenuated increased GBM cell respiration facilitated by panobinostat. What follows is the activation of an integrated stress response with characteristic activation of eif2α signaling, culminating in an increase of the stress response transcription factor, ATF4 [33,34,35]. Although our experiments did not directly connect these two phenomena, previous work by us and others has demonstrated a tight linkage between energy deprivation (here mediated by dual loss of the major energetic pathways) and the activation of the integrated stress response [36]. In part, loss of ATP is linked due to accumulation of unfolded proteins given that proper maturation and protein synthesis depend on an appropriate state of energy within cells and altering this rheostat will affect the integrity of tumor cells [37].

The integrated stress response mediators ATF4 and ATF3 have been known to impact tumor cell survival in part through regulation of the Bcl-2 family members of proteins [16,17]. Our silencing experiments demonstrated the involvement of Noxa in cell death elicited by the combination treatment, which is in keeping with certain other drug combination treatments [38,39,40,41]. In addition, we validated the involvement of pro-apoptotic BAX and BAK in the drug combination through siRNA experiments and it seems that BAK is particularly important for the combination treatment to exert its effects. These results are in keeping with other studies that have demonstrated involvement of the Bcl-2 family of proteins in cell killing since usually BAX and BAK are necessary for cell death induction under such circumstances [38,39,40,41]. Although we have not pursued the approach of simultaneous inhibition of both BAX/BAK, this strategy oftentimes results in an even stronger suppression of cell death induction by certain stimuli [42]. Classically, BH3-mimetics would be expected to be particularly prone to this strategy since their mechanism of action depends on the release of BAX and BAK from either Bcl-2 and/or Bcl-xL [43,44]. Our finding that in GBM14 the drug combination of gamitrinib and romidespin elicited an increase of Bcl-2 is intriguing when this observation is put into the context of BH3-mimetics, especially the FDA-approved drug, venetoclax (ABT-199). It would be tempting to speculate that presumably ABT-199 might further enhance the efficacy of the drug combination of gamitrinib and romidepsin. Future studies may address such an approach further.

We also confirmed that the combination treatment acted in PDX models of human GBM [3] to reduce tumor growth without any major toxicity event. These results are reassuring in that other drug combinations, involving gamitrinib, have performed reasonably well in terms of efficacy, while at the same time displaying minimal toxicity. For instance, we have recently demonstrated that the combination treatment of gamitrinib and the BH3-mimetic, ABT263, has extended overall survival in an orthotopic PDX model of GBM in mice [45]. Similarly, we found that activation of LXR receptors along with interference of TRAP1 led to enhanced growth reduction of heterotopic xenograft of GBM in mice [46]. Other groups have demonstrated that gamitrinib enhanced the efficacy of BRAF-inhibitors in model systems of melanoma in part by the ability of gamitrinib to dampen tumor cell respiration [47]. How efficient gamitrinib and/or panobinostat penetrate the blood brain barrier (BBB) has not formally been documented. However, given that brain tumors disrupt the BBB, it is likely that at the very least a significant fraction of glial tumor cells may be exposed to the drugs. However, additional more detailed liquid chromatography/mass spectrometry (LC/MS) studies would be necessary to determine the degree of penetration.

## 5. Conclusions

Combined inhibition of TRAP1 and HDACs is a potential novel strategy to combat recalcitrant malignancies, such as GBM. We have unraveled a mechanism that relates to tumor cell metabolism, the integrated stress response and modulation of Bcl-2 family members, which all in all resulted in the induction of a caspase dependent cell death with apoptotic features.

## Figures and Tables

**Table 1:** Primers for real time PCR.

Gene	Forward Sequence	Reserve Sequence
GRP78 (HSPA5)	CTGTCCAGGCTGGTGTGCTCT	CTTGGTAGGCACCACTGTGTTC
Noxa (PMAIP1)	CTGGAAGTCGAGTGTGCTACTC	TGAAGGAGTCCCCTCATGCAAG
XBP1	CTGCCAGAGATCGAAAGAAGGC	CTCCTGGTTCTCAACTACAAGGC
CHOP (DDIT3)	GGTATGAGGACCTGCAAGAGGT	CTTGTGACCTCTGCTGGTTCTG
CEBPB	AGAAGACCGTGGACAAGCACAG	CTCCAGGACCTTGTGCTGCGT
ATF3	CGCTGGAATCAGTCACTGTCAG	CTTGTTTCGGCACTTTGCAGCTG
ATF4	TTCTCCAGCGACAAGGCTAAGG	CTCCAACATCCAATCTGTCCCG

## Supplementary Materials

The following are available online at https://www.mdpi.com/2073-4409/9/7/1661/s1, Figure S1. Combined treatment with gamitrinib and HDAC inhibitors elicits synergistic reduction in cellular proliferation of GBM cells, Figure S2. Combined inhibition of TRAP1 and HDACs enhanced cell death in GBM cells, Figure S3. Combined inhibition of TRAP1 and HDACs enhanced cell death in GBM cells, Figure S4. Combined inhibition of TRAP1 and HDACs enhanced activation of a cell death with apoptotic features, including cleavage of caspases, Figure S5. The combination treatment of gamitrinib and HDAC inhibitors modulates the expression of the Bcl-2 family of proteins in GBM cells, Figure S6. The combination treatment of gamitrinib and HDAC inhibitors interferes with GBM cell OXPHOS (oxidative phosphorylation) complexes, Figure S7. Combined treatment with gamitrinib (GTPP) and HDAC inhibitor, panobinostat, elicits no detectable organ toxicity in vivo. Supplementary Whole Blot: The whole blot showing all the bands with all molecular weight markers on the Western.
