# Supplementary material for: Inhibition of HDAC1/2 Along with TRAP1 Causes Synthetic Lethality in Glioblastoma Model Systems
Source: Cells. 2020 Jul 10;9(7):1661. doi: 10.3390/cells9071661 (PMC7407106; doi:10.3390/cells9071661)
Supplement: Supplementary file 1 [file cells-09-01661-s001.pdf]

cells

Supplementary Materials

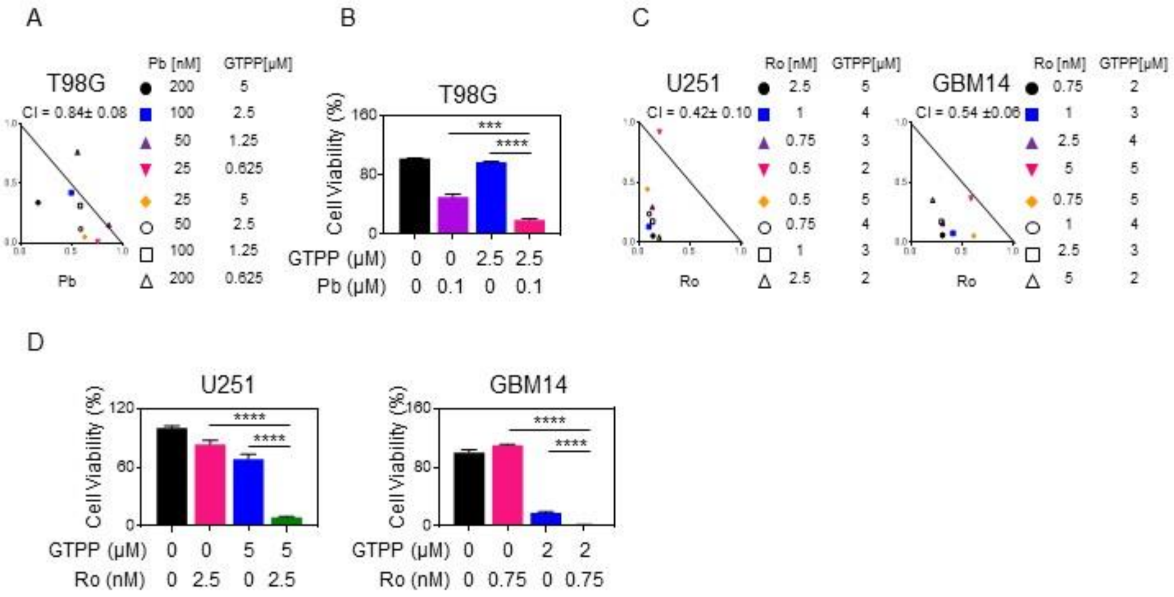

Figure S1. Combined treatment with gamitrinib and HDAC inhibitors elicits synergistic reduction in cellular proliferation of GBM cells. (**A**) T98G cells were treated with gamitrinib (GTPP) and panobinostat or the combination of both for 72h. Thereafter, cellular viability and statistical analysis were performed. Isobolograms are shown; (**B**) The graph shows cellular viability data following

treatment with vehicle, panobinostat, gamitrinib or the combination for 72h in the indicated GBM cells (n=3); (**C**) U251 and GBM14 cells were treated with gamitrinib and romidepsin for 72h. Thereafter, cellular viability and statistical analysis were performed. Isobolograms are shown; (**D**) The graphs show cellular viability data following treatment with vehicle, romidepsin, gamitrinib or the combination for 72h in the indicated GBM cells (n=4). Shown are means and SD. ANOVA was used for statistical analysis. ****$p<0.001$.

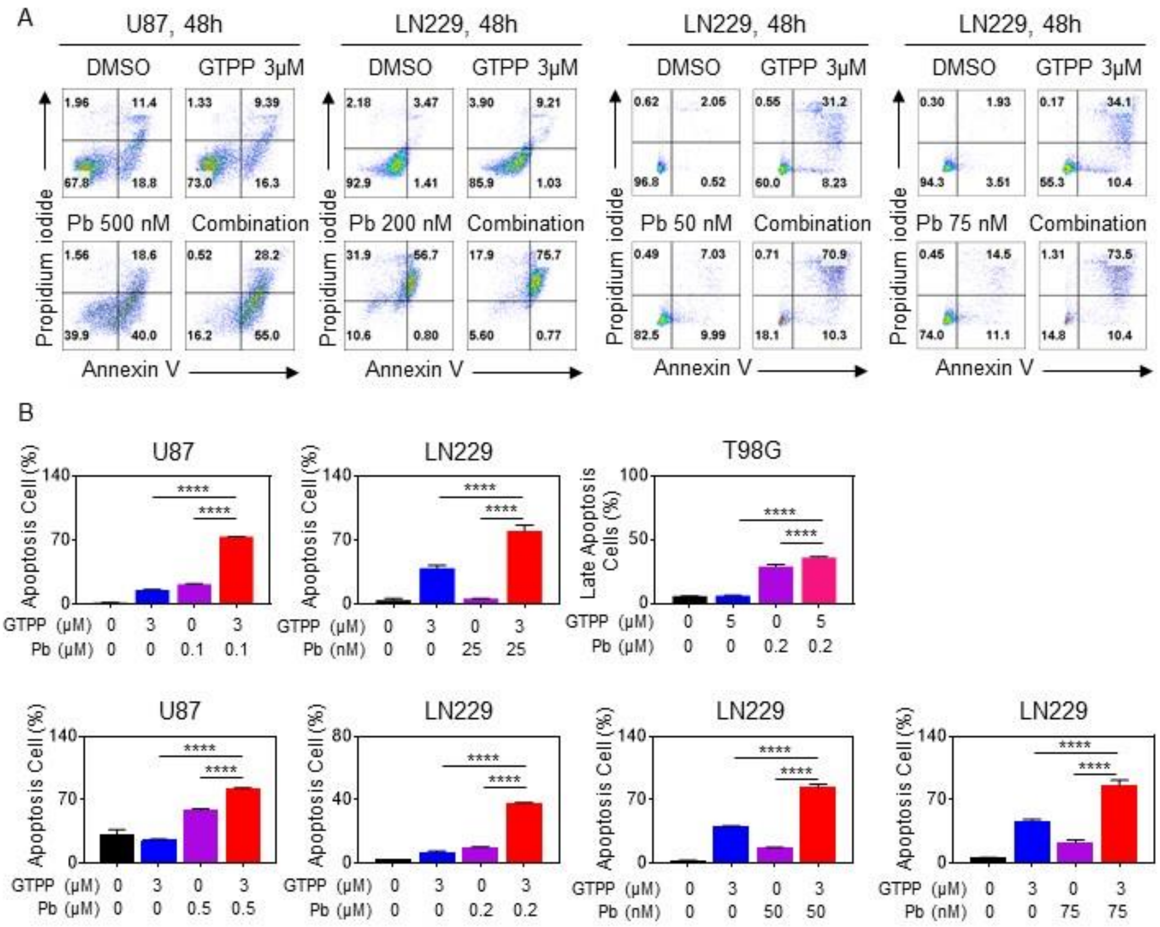

Figure S2. Combined inhibition of TRAP1 and HDACs enhanced cell death in GBM cells. (**A**) U87 and LN229 cells were treated with the indicated concentration of gamitrinib, panobinostat, or the combination of both for 48h. Thereafter, cells were labeled with annexin/propidium iodide (PI) dye and analyzed by multi-parametric flow cytometry. Shown are representative flow plots; (**B**) The graphs show apoptotic cells of gamitrinib, panobinostat, or the combination of both in U87, LN229, and T98G (n=3). Shown are means and SD. ANOVA was used for statistical analysis. ****$p<0.001$.

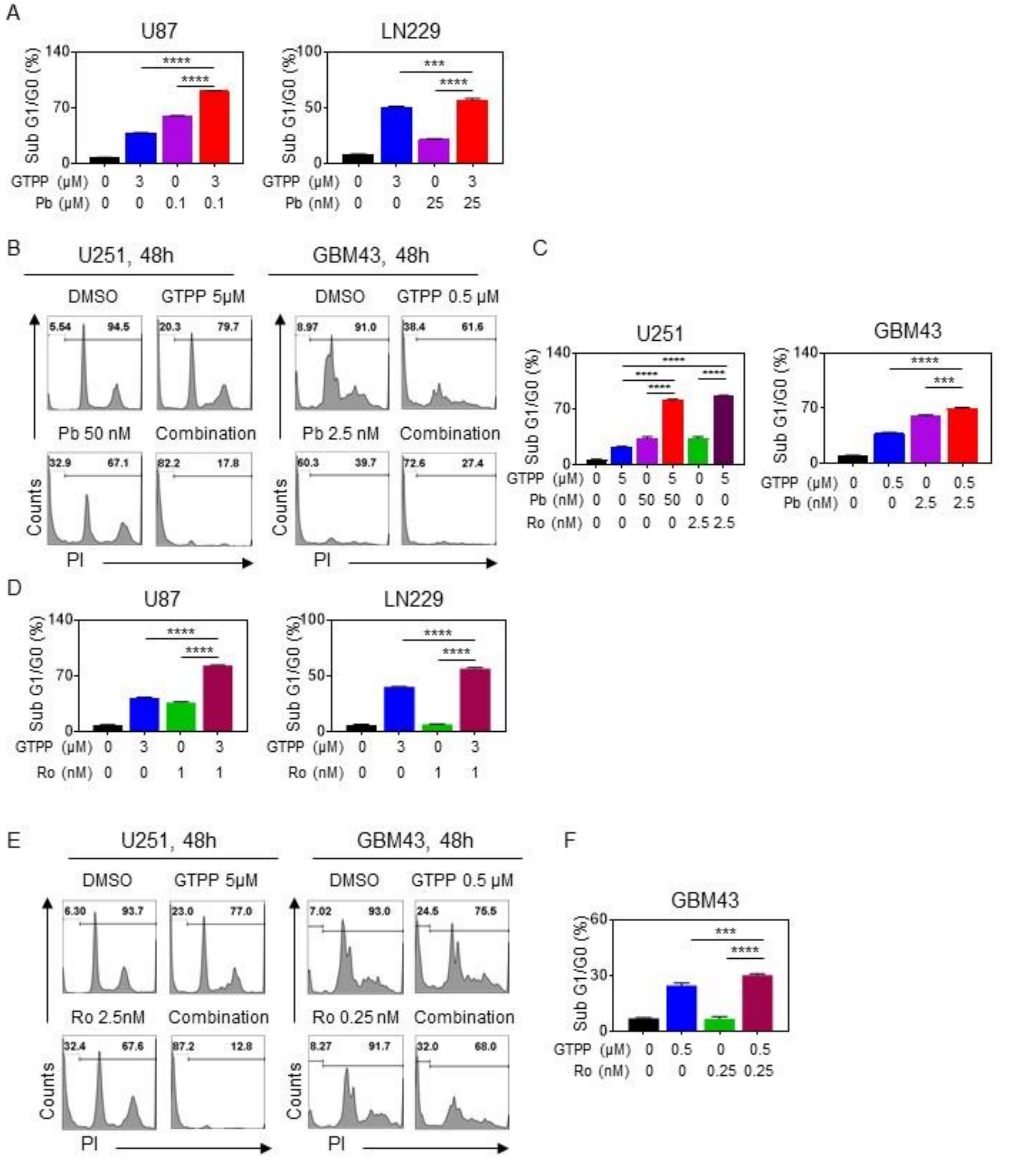

Figure S3. Combined inhibition of TRAP1 and HDACs enhanced cell death in GBM cells. (**A**) The graphs show sub G1/G0 of U87 and LN229 cells treated with the indicated concentrations of gamitrinib and panobinostat (n=3); (**B**) U251 and GBM43 cells were treated with the indicated concentration of gamitrinib, panobinostat or combination of both for 48h. Thereafter, cells were labeled with propidium iodide (PI) dye and analyzed by flow cytometry. Shown are representative flow plots; (**C**) The graphs show sub G1/G0 of U251 and GBM43 cells treated with the indicated

concentrations of gamitrinib and panobinostat/romidepsin (n=3); (**D**) The graphs show sub G1/G0 of U87 and LN229 cells were treated with the indicated concentration of gamitrinib, romidepsin, or combination of both (n=3); (**E**) U251 and GBM43 cells were treated with the indicated concentration of gamitrinib, romidepsin, or combination of both for 48h. Thereafter, cells were labeled with propidium iodide (PI) dye and analyzed by flow cytometry. Shown are representative flow plots; (**F**) The graph shows sub G1/G0 of GBM43 cells treated with the indicated concentrations of gamitrinib, romidepsin, or combination of both (n=3). Shown are means and SD. ANOVA was used for statistical analysis. ***/****p<0.001.

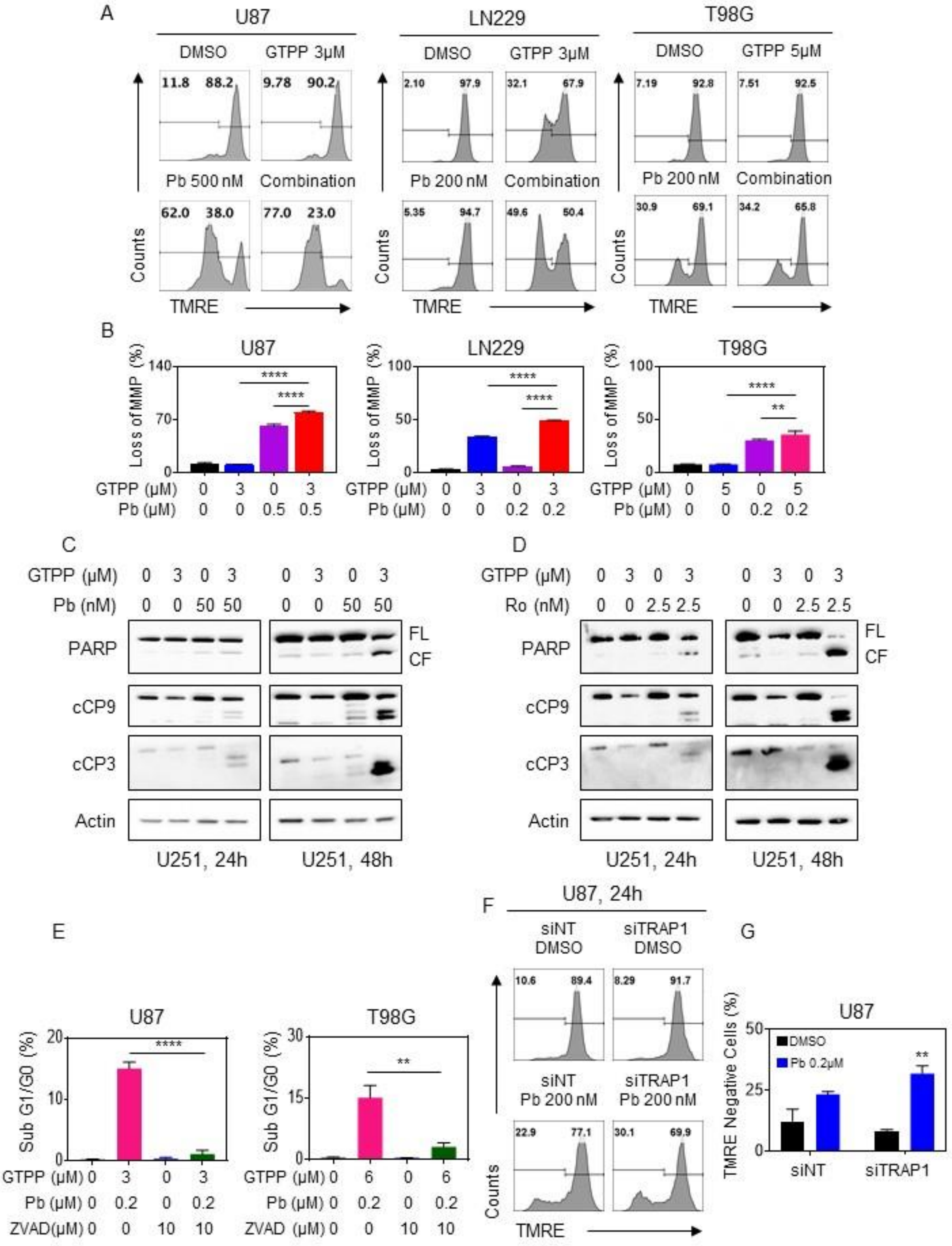

Figure S4. Combined inhibition of TRAP1 and HDACs enhanced activation of a cell death with apoptotic features, including cleavage of caspases. (**A, B**) U87, LN229, and T98G cells were treated with the indicated concentration of gamitrinib, panobinostat, or combination of both. Thereafter, cells were labeled with TMRE dye and analyzed by flow cytometry (n=3). Shown are means and SD. ANOVA was used for statistical analysis; (**C, D**) Standard western blots of cell lysates of U251 treated with gamitrinib, panobinostat/romidepsin, or the combination of both for 24h and 48h. Actin is used

as a loading control. FL: full length, CF: cleaved fragment; (**E**) The graphs show sub G1/G0 of U87 and T98G cells treated with the indicated concentrations of gamitrinib and panobinostat in the presence or absence of zVAD (n=3); (**F, G**) U87 cells were transfected with non-targeting (siNT) or TRAP1 specific siRNA (siTRAP1) and following 48h treated with panobinostat for 24h. Thereafter, cells were labeled with TMRE dye and analyzed by flow cytometry (n=3). Shown are means and SD. Statistical significance was determined by two-tailed Student's t-test. $^{**}p<0.01$, $^{****}p<0.001$.

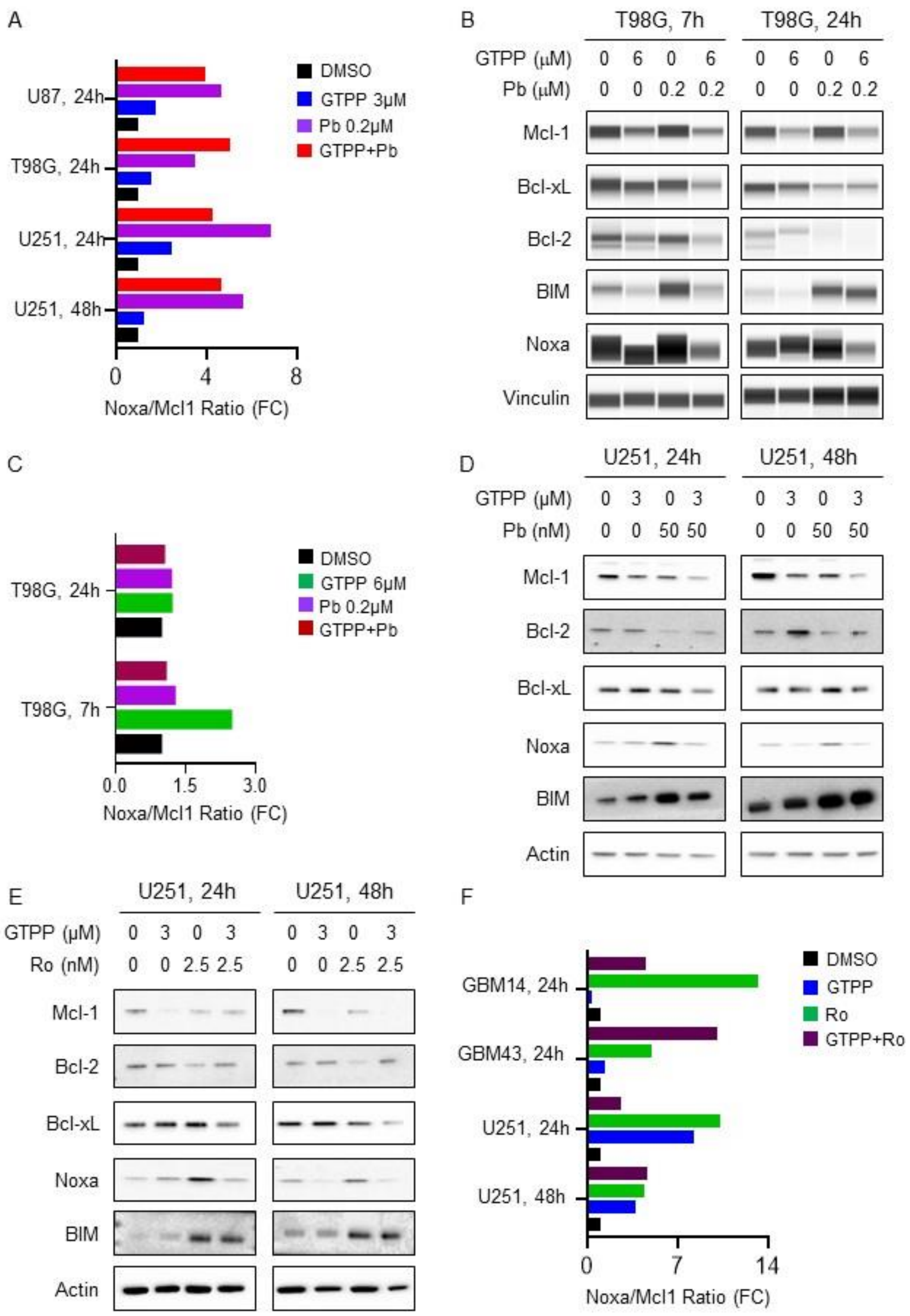

Figure S5. The combination treatment of gamitrinib and HDAC inhibitors modulates the expression of the Bcl-2 family of proteins in GBM cells. (**A**) The graph shows the Noxa/Mcl1 ratio in U87, T98G, and U251 cells treated with gamitrinib, panobinostat, or the combination of both; (**B**) T98G cells were treated with gamitrinib, panobinostat, or the combination for 7h and 24h. The whole cell lysates were subjected to protein capillary electrophoresis. Vinculin serves as loading control; (**C**) The graph shows the Noxa/Mcl1 ratio in T98G cells treated with gamitrinib, panobinostat, or the combination

of both; (**D, E**) Standard western blots of cell lysates of U251 treated with gamitrinib, panobinostat/romidepsin, or the combination of both for 24h and 48h. Actin is used as a loading control; (**F**) The graph shows the Noxa/Mcl1 ratio in GBM14, GBM43, and U251 cells treated with gamitrinib, romidepsin, or the combination of both.

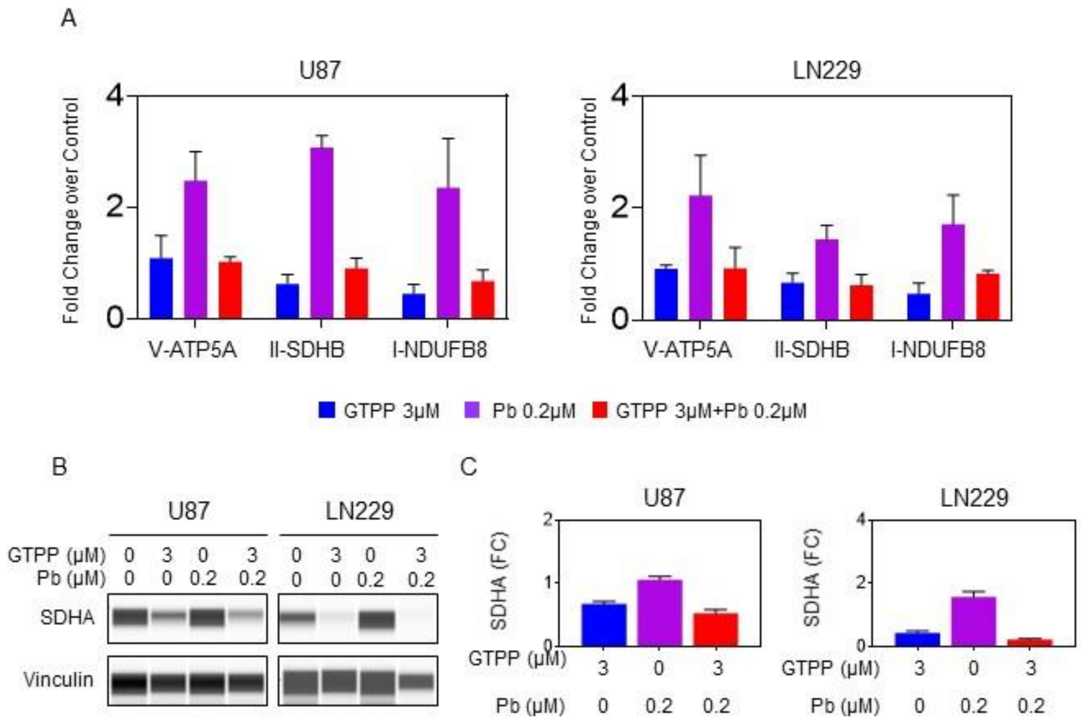

Figure S6. The combination treatment of gamitrinib and HDAC inhibitors interferes with GBM cell OXPHOS (oxidative phosphorylation) complexes. (**A**) The graphs show the expression of OXPHOS

complex in U87 and LN229 cells treated with with gamitrinib, panobinostat, or the combination of both for 24h (n=2); (**B, C**) U87 and LN229 cells were treated with gamitrinib, panobinostat, or the combination for 24h. The whole cell lysates were subjected to protein capillary electrophoresis. Vinculin serves as loading control (n=3). Shown are means and SD (fold change (FC) over control).

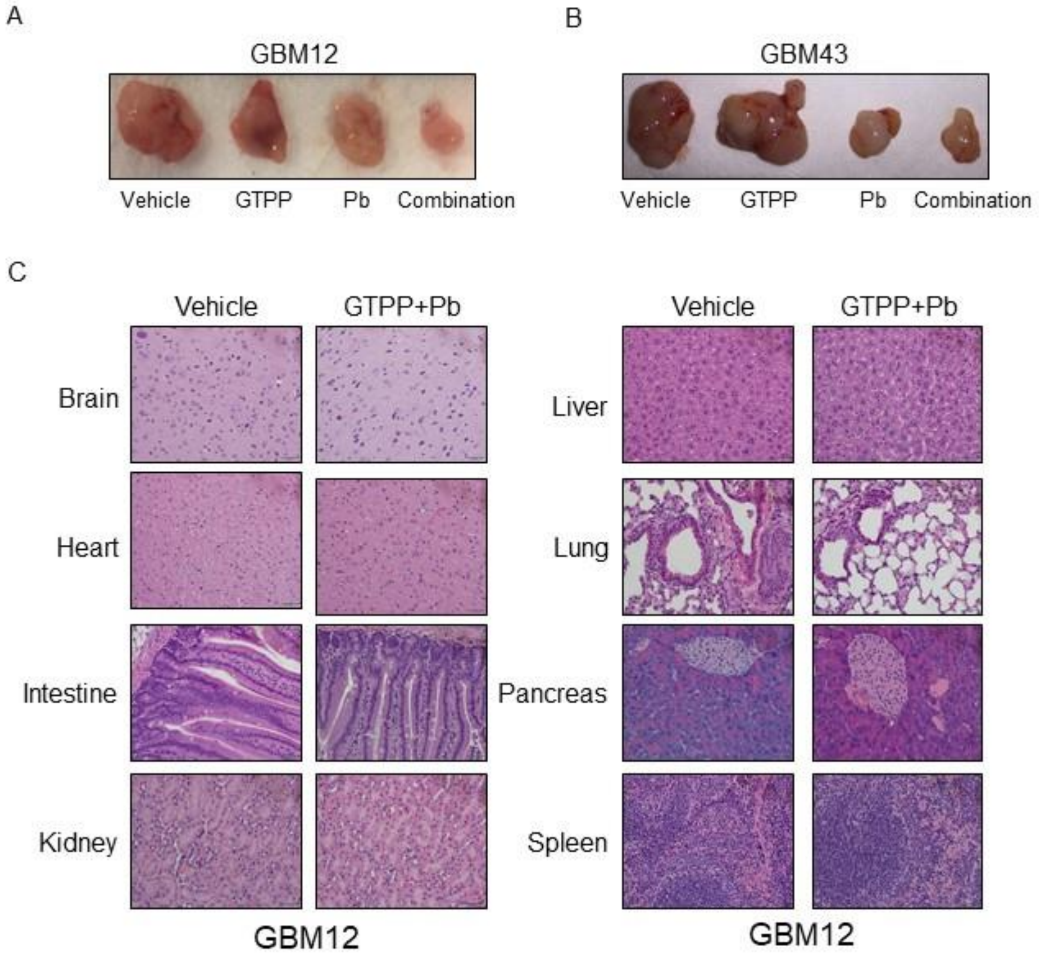

Figure S7. Combined treatment with gamitrinib (GTPP) and HDAC inhibitor, panobinostat, elicits no detectable organ toxicity in vivo. (**A, B**) GBM12 and GBM43 cells were implanted into the subcutis

of immunocompromised Nu/Nu mice. After the tumors were established, randomization was assigned to four treatment groups: vehicle, gamitrinib (3 mg/kg), panobinostat (5 mg/kg), and combination treatment of both. Shown are the tumor of four treatment groups on the last day; (**C**) Shown is the H&E staining of the vehicle and gamitrinib and panobinostat combination treatment in different organelle of GBM12 cells in (**A**).

Uncropped blot and capillary electrophoresis images

Figure 2D

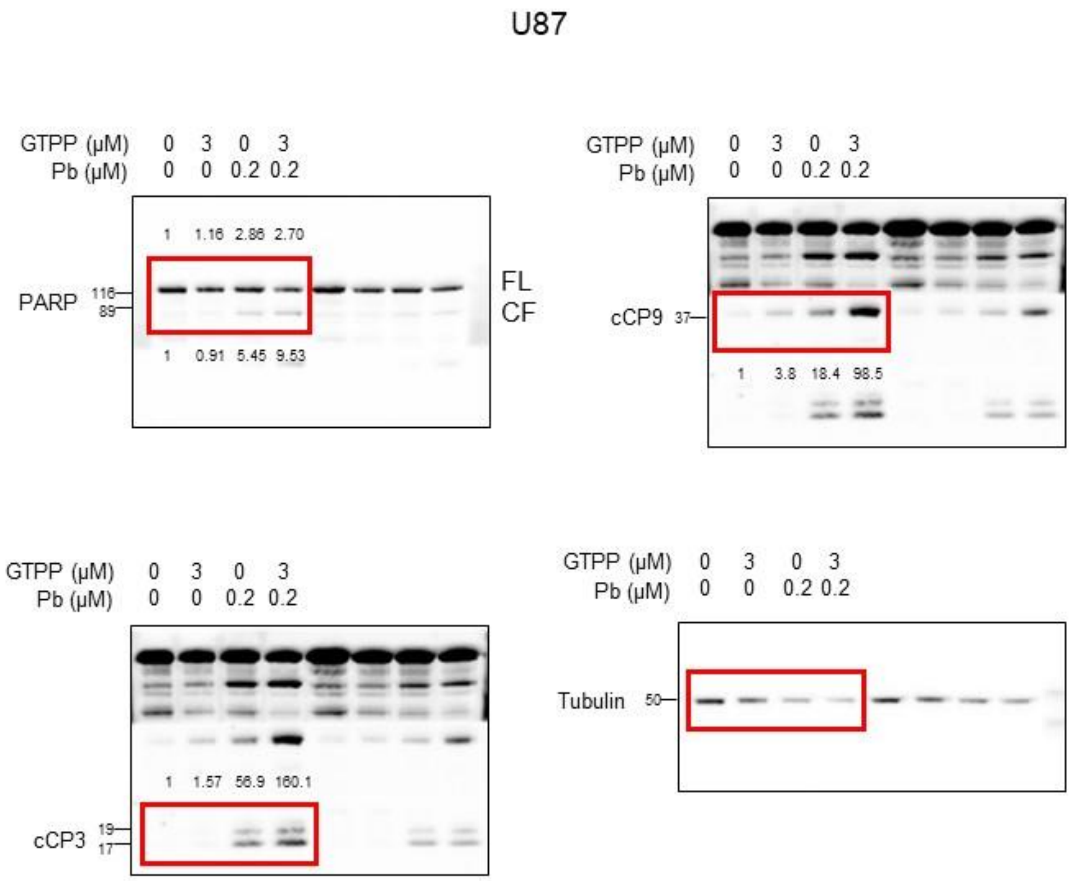
U87
GTPP (μM) 0 3 0 3
Pb (μM) 0 0 0.2 0.2
1 1.16 2.86 2.70
PARP 116 89
FL
CF
1 0.91 5.45 9.53
GTPP (μM) 0 3 0 3
Pb (μM) 0 0 0.2 0.2
cCP9 37
1 3.8 18.4 98.5
GTPP (μM) 0 3 0 3
Pb (μM) 0 0 0.2 0.2
1 1.57 56.9 160.1
cCP3 19 17
GTPP (μM) 0 3 0 3
Pb (μM) 0 0 0.2 0.2
Tubulin 50

Figure 2D

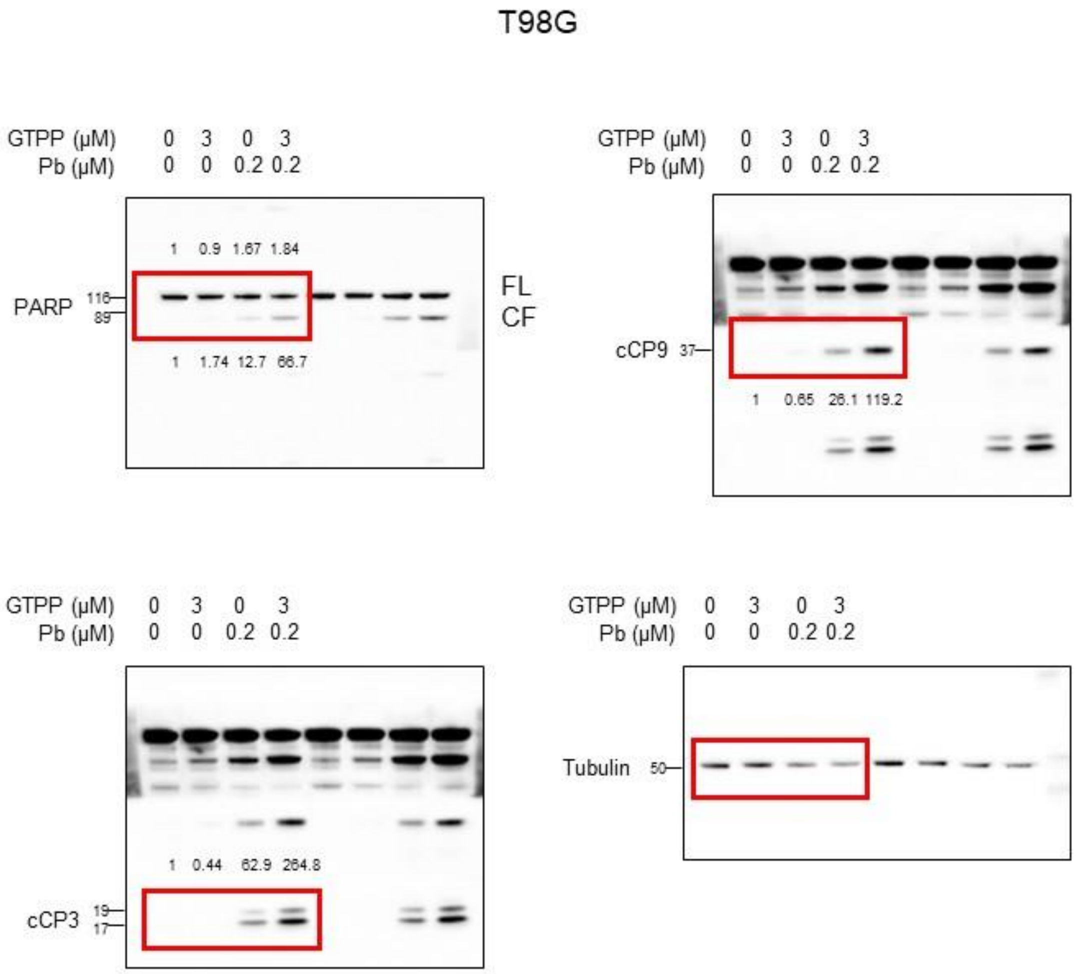
T98G
GTPP (μM) 0 3 0 3
Pb (μM) 0 0 0.2 0.2
1 0.9 1.67 1.84
PARP 116 89
FL
CF
1 1.74 12.7 66.7
GTPP (μM) 0 3 0 3
Pb (μM) 0 0 0.2 0.2
cCP9 37
1 0.65 26.1 119.2
GTPP (μM) 0 3 0 3
Pb (μM) 0 0 0.2 0.2
1 0.44 62.9 264.8
cCP3 19 17
GTPP (μM) 0 3 0 3
Pb (μM) 0 0 0.2 0.2
Tubulin 50

Figure 2G

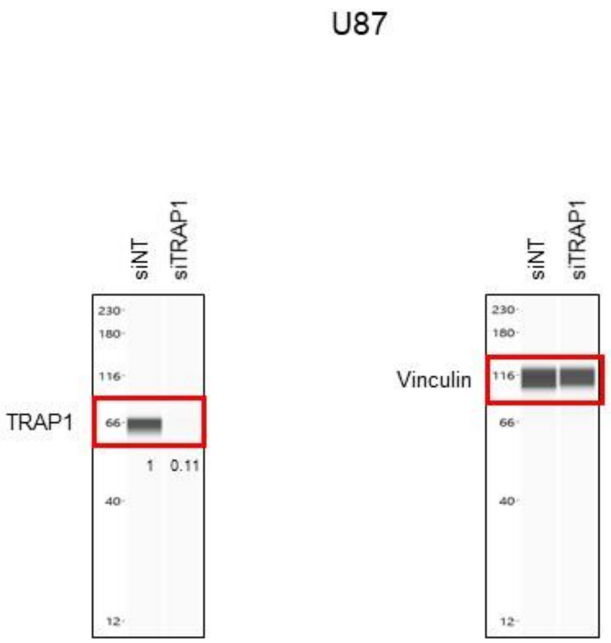

78

Figure 3A

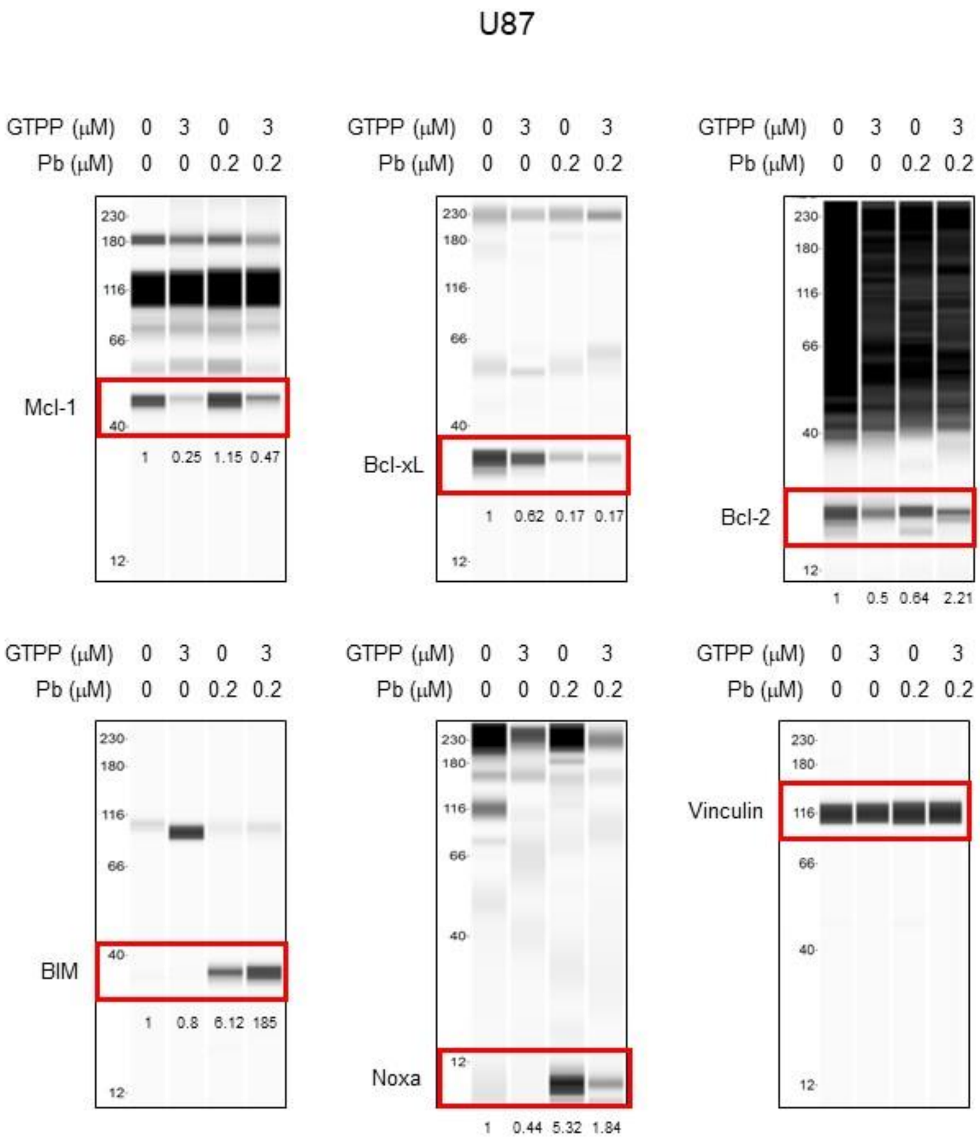
U87
GTPP (μM) 0 3 0 3
Pb (μM) 0 0 0.2 0.2
Mcl-1
1 0.25 1.15 0.47
Bcl-xL
1 0.62 0.17 0.17
Bcl-2
1 0.5 0.64 2.21
BIM
1 0.8 6.12 185
Noxa
1 0.44 5.32 1.84
Vinculin
230
180
116
66
40
12

Figure 3A

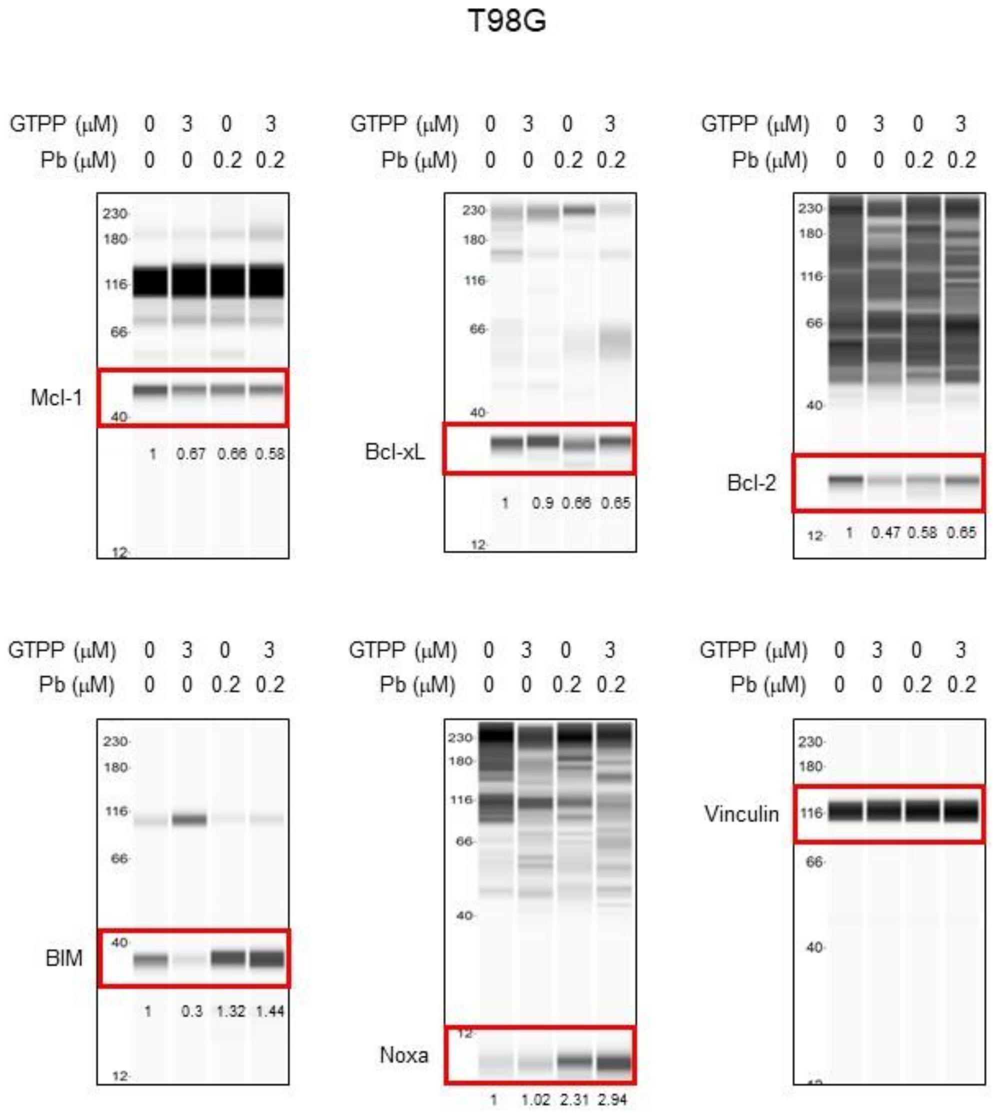
T98G
GTPP (µM) 0 3 0 3
Pb (µM) 0 0 0.2 0.2
Mcl-1
1 0.67 0.66 0.58
Bcl-xL
1 0.9 0.66 0.65
Bcl-2
1 0.47 0.58 0.65
BIM
1 0.3 1.32 1.44
Noxa
1 1.02 2.31 2.94
Vinculin
230
180
116
66
40
12

Figure 3B

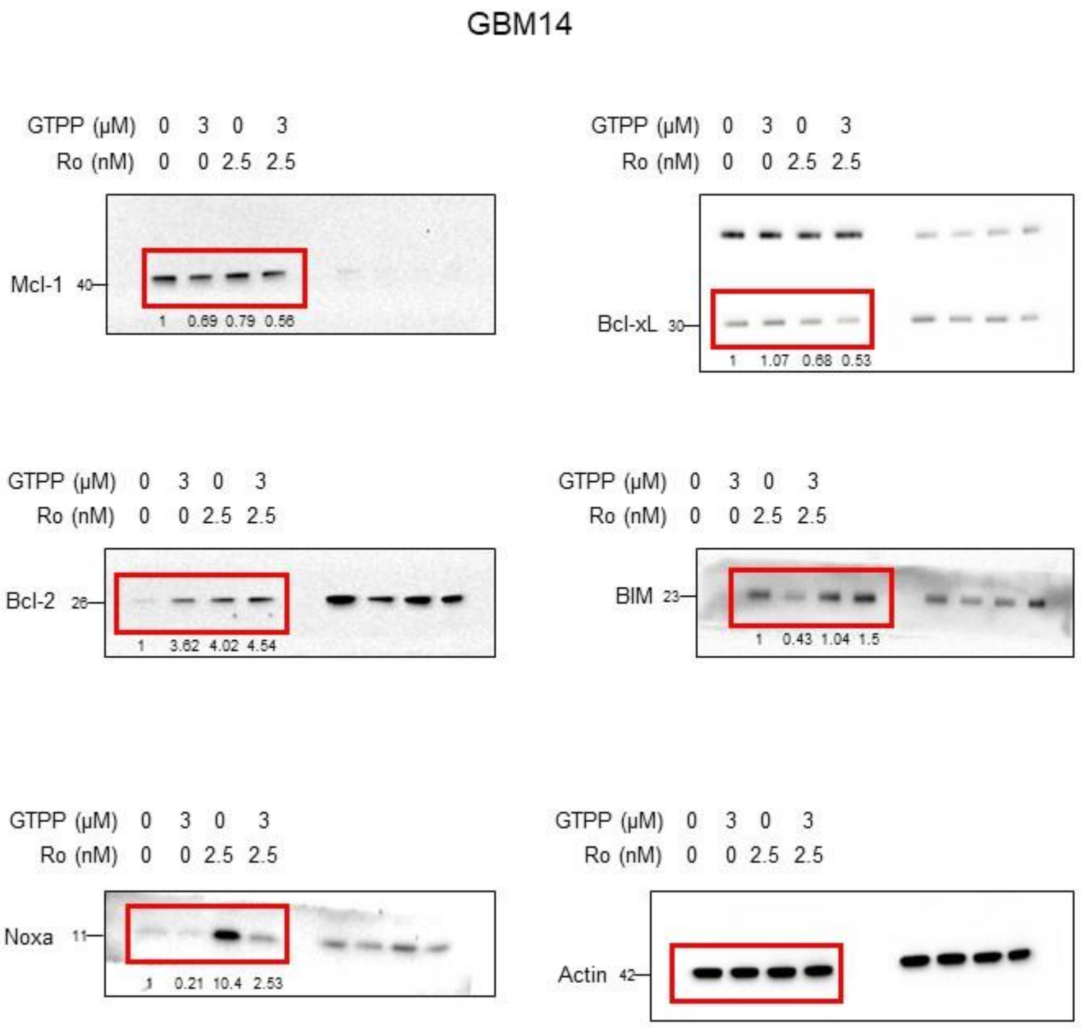
GBM14
GTPP (μM) 0 3 0 3
Ro (nM) 0 0 2.5 2.5
Mcl-1 40
1 0.69 0.79 0.56
Bcl-xL 30
1 1.07 0.68 0.53
Bcl-2 26
1 3.62 4.02 4.54
BIM 23
1 0.43 1.04 1.5
Noxa 11
1 0.21 10.4 2.53
Actin 42

Figure 3B

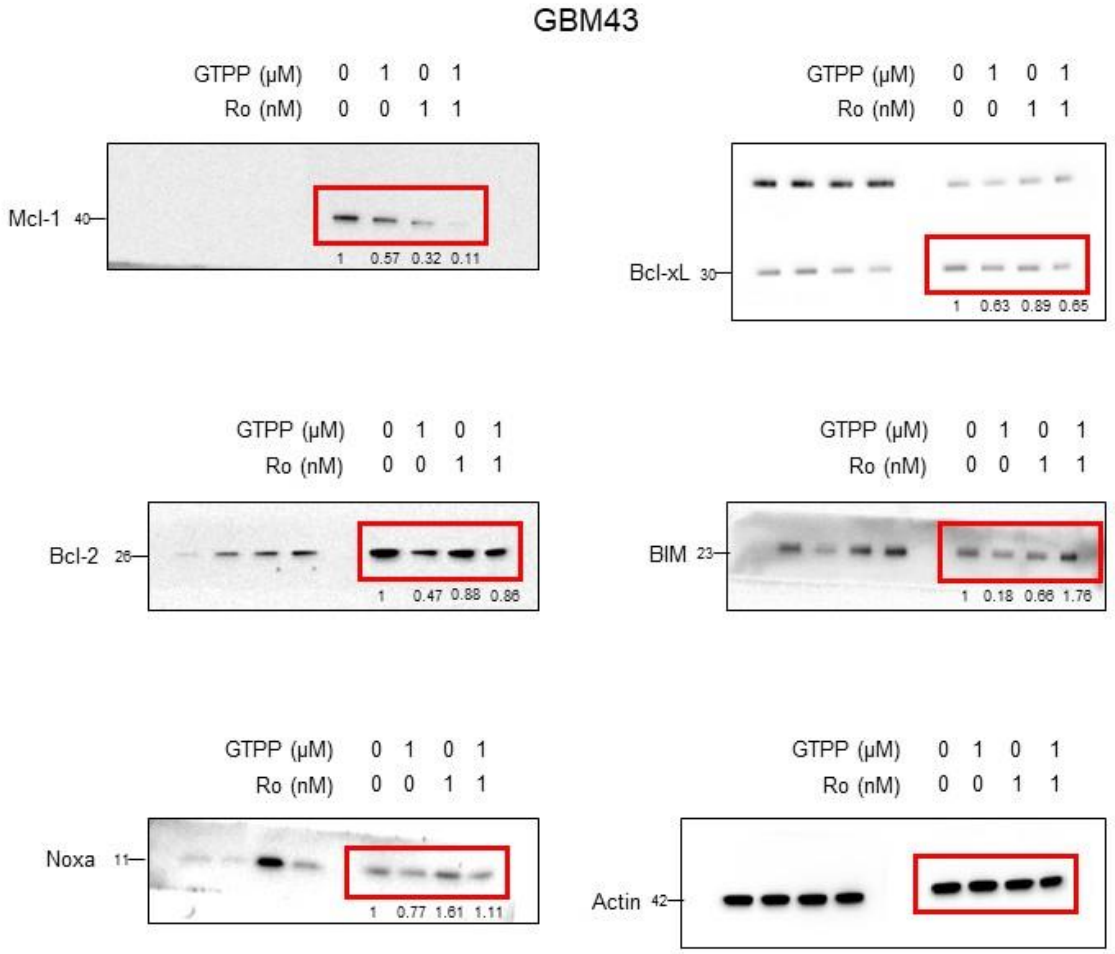
GBM43
GTPP (μM) 0 1 0 1
Ro (nM) 0 0 1 1
Mcl-1 40
1 0.57 0.32 0.11
Bcl-xL 30
1 0.63 0.89 0.65
Bcl-2 26
1 0.47 0.88 0.86
BIM 23
1 0.18 0.66 1.76
Noxa 11
1 0.77 1.61 1.11
Actin 42

Figure 3D

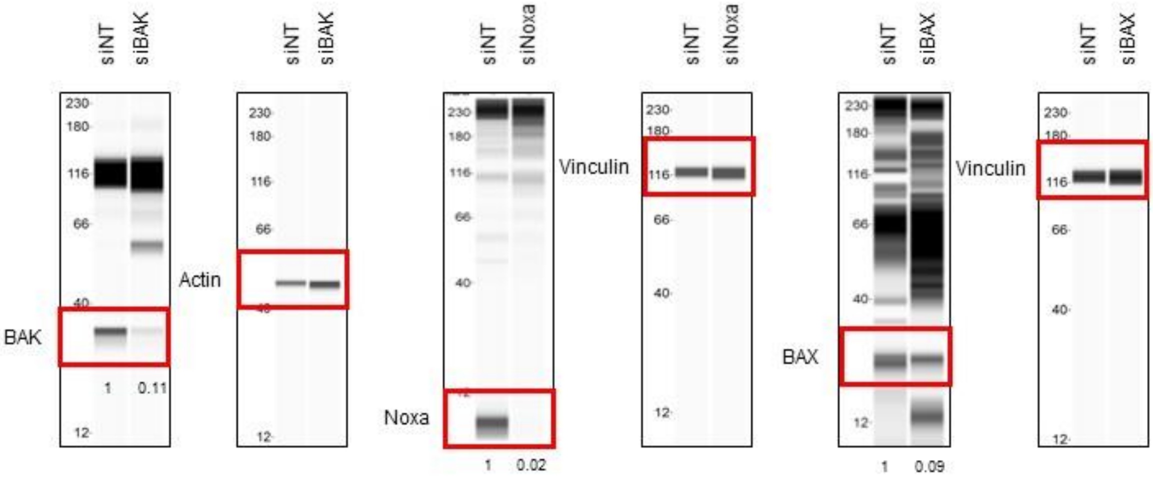
siNT
siBAK
BAK
1
0.11
Actin
siNoxa
Noxa
0.02
Vinculin
siBAX
BAX
0.09
230
180
116
66
40
12

83

Figure 4A

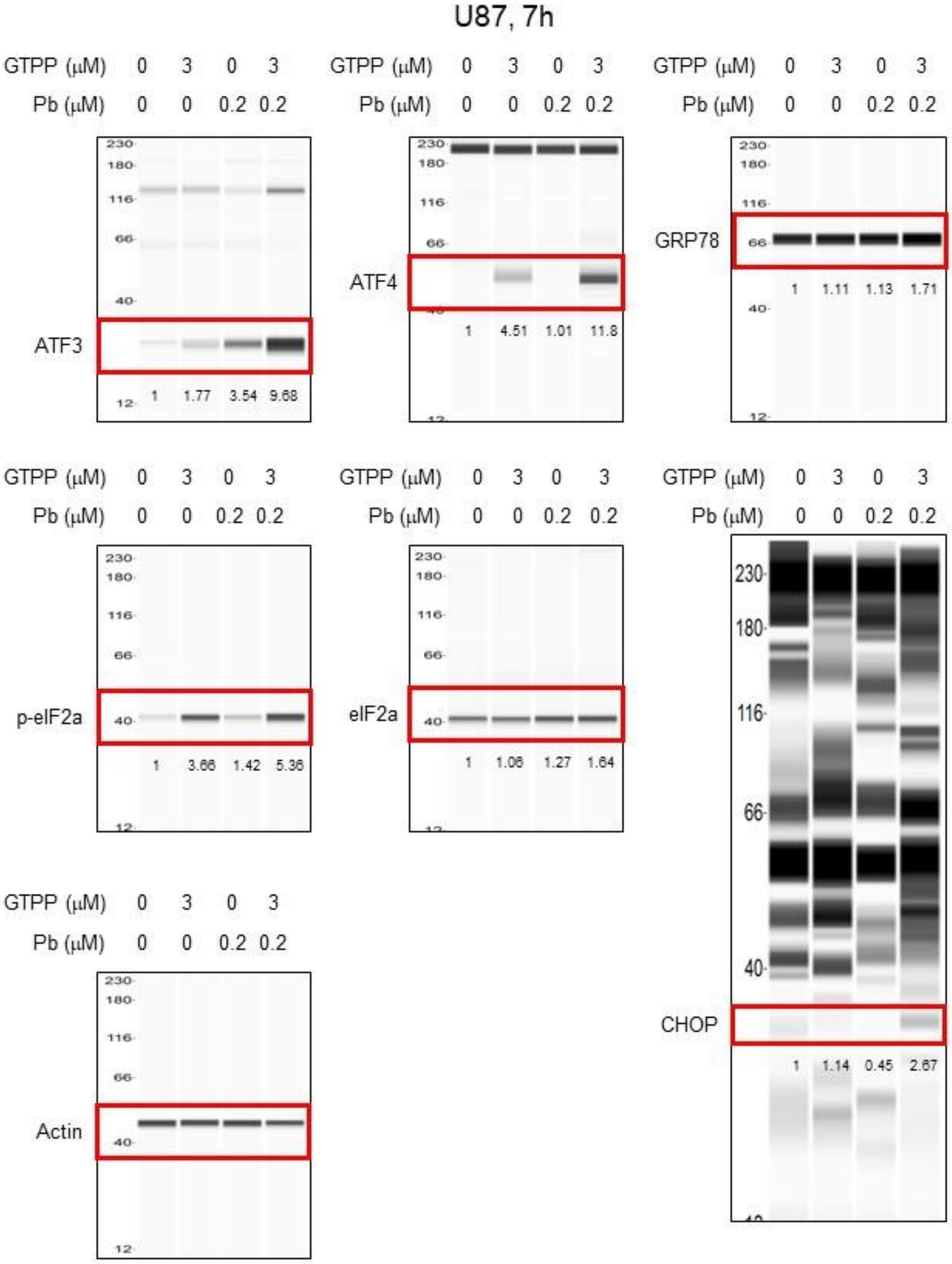
U87, 7h
GTPP (μM) 0 3 0 3
Pb (μM) 0 0 0.2 0.2
ATF3
1 1.77 3.54 9.68
ATF4
1 4.51 1.01 11.8
GRP78
1 1.11 1.13 1.71
p-eIF2a
1 3.66 1.42 5.36
eIF2a
1 1.06 1.27 1.64
CHOP
1 1.14 0.45 2.67
Actin
230
180
116
66
40
12

84

Figure 4A

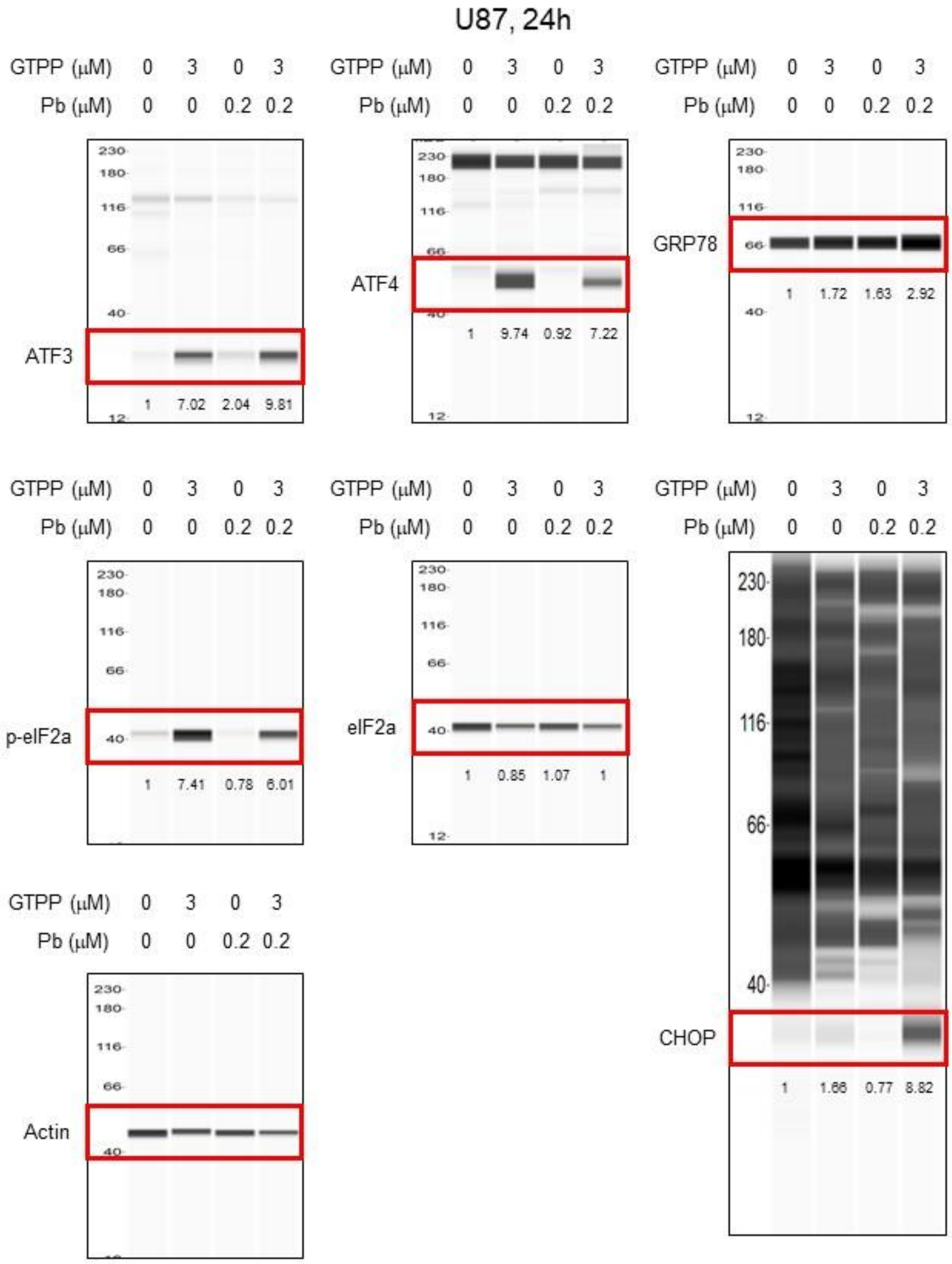
U87, 24h
GTPP (μM) 0 3 0 3
Pb (μM) 0 0 0.2 0.2
ATF3
1 7.02 2.04 9.81
ATF4
1 9.74 0.92 7.22
GRP78
1 1.72 1.63 2.92
p-elF2a
1 7.41 0.78 6.01
elF2a
1 0.85 1.07 1
CHOP
1 1.66 0.77 8.82
Actin
230
180
116
66
40
12

85

Figure 4A

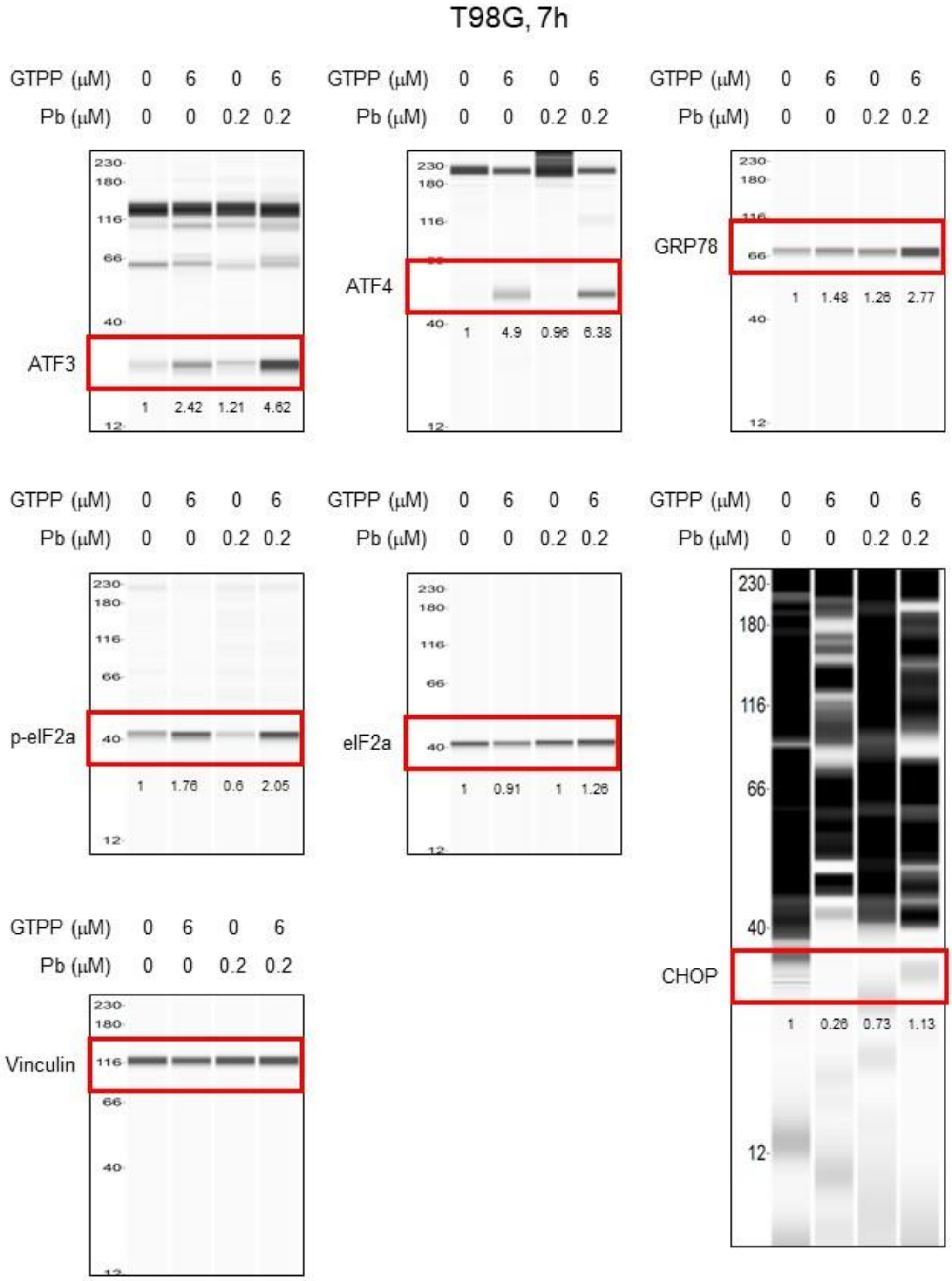
T98G, 7h
GTPP (μM) 0 6 0 6
Pb (μM) 0 0 0.2 0.2
ATF3
1 2.42 1.21 4.62
ATF4
1 4.9 0.96 6.38
GRP78
1 1.48 1.26 2.77
p-eIF2a
1 1.76 0.6 2.05
eIF2a
1 0.91 1 1.26
CHOP
1 0.26 0.73 1.13
Vinculin
230
180
116
66
40
12

86

Figure 4A

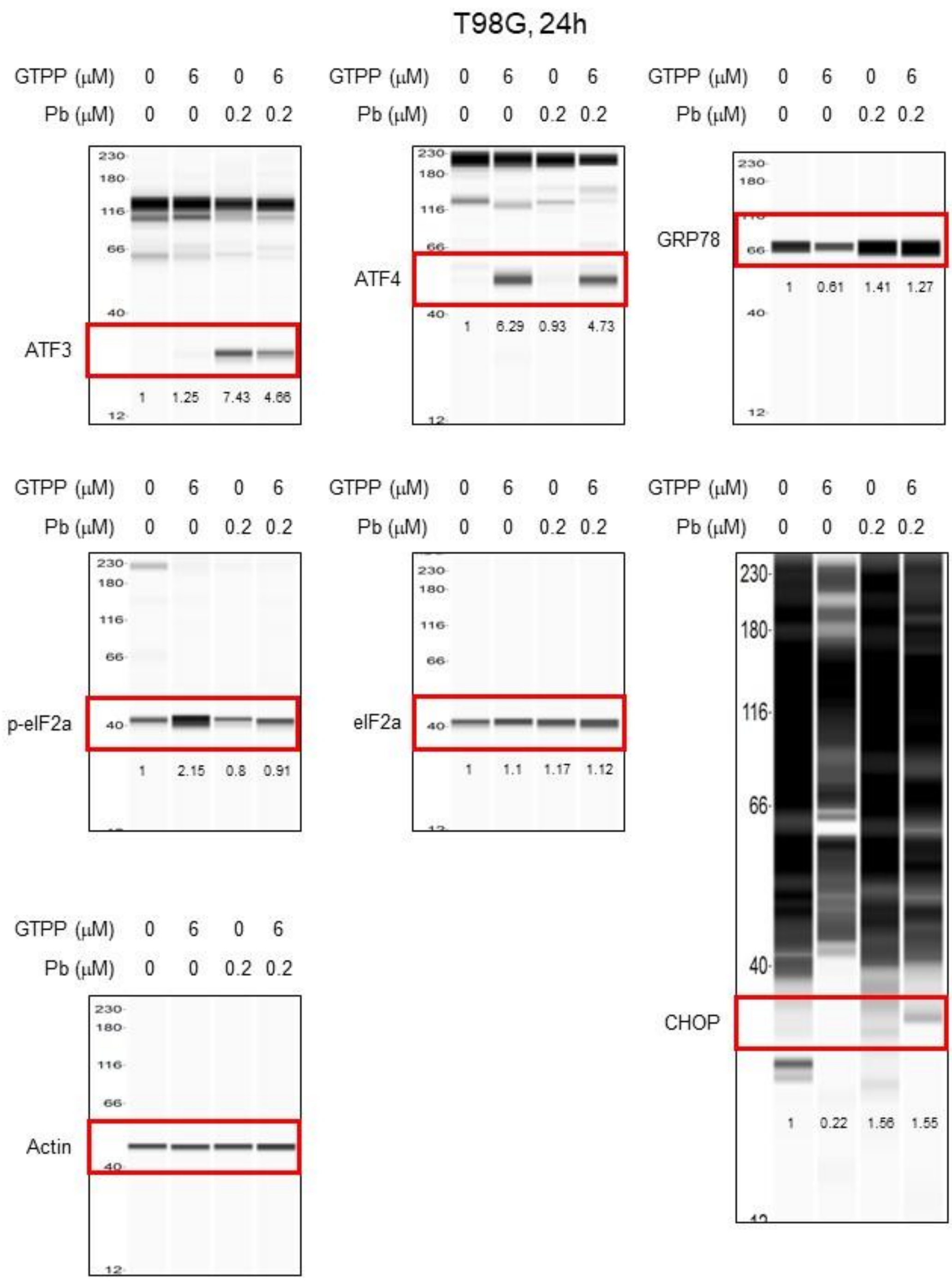
T98G, 24h
GTPP (µM) 0 6 0 6
Pb (µM) 0 0 0.2 0.2
230
180
116
66
40
12
ATF3
1 1.25 7.43 4.66
ATF4
1 6.29 0.93 4.73
GRP78
1 0.61 1.41 1.27
p-eIF2a
1 2.15 0.8 0.91
eIF2a
1 1.1 1.17 1.12
CHOP
1 0.22 1.56 1.55
Actin

Figure 5D

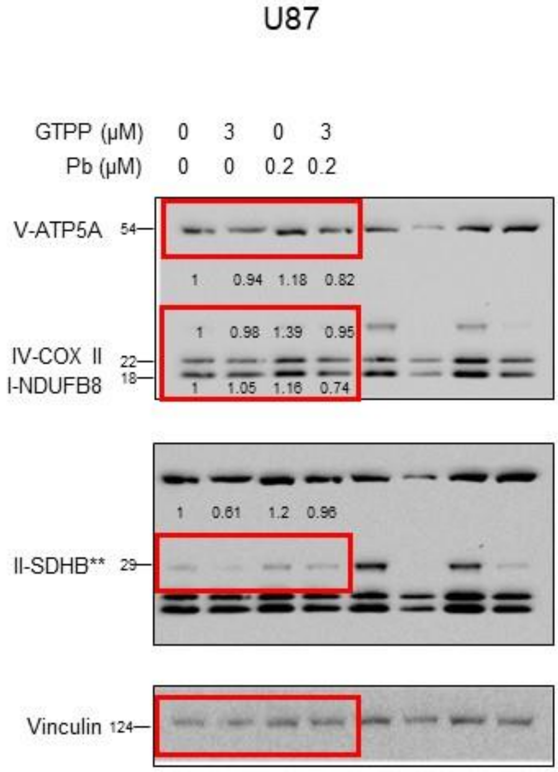
U87
GTPP (μM) 0 3 0 3
Pb (μM) 0 0 0.2 0.2
V-ATP5A
54
1 0.94 1.18 0.82
1 0.98 1.39 0.95
IV-COX II
22
I-NDUFB8
18
1 1.05 1.16 0.74
1 0.61 1.2 0.96
II-SDHB**
29
Vinculin
124

88

Figure 5D

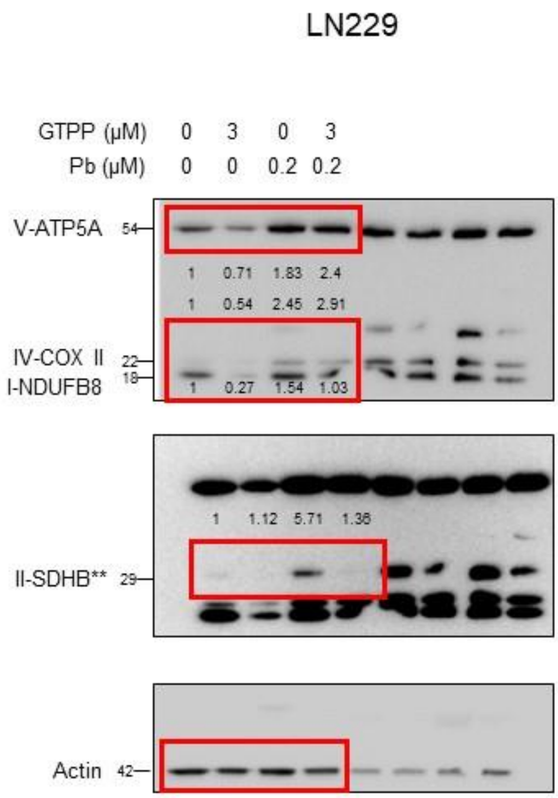
LN229
GTPP (μM) 0 3 0 3
Pb (μM) 0 0 0.2 0.2
V-ATP5A
54
1 0.71 1.83 2.4
1 0.54 2.45 2.91
IV-COX II
I-NDUFB8
22
18
1 0.27 1.54 1.03
1 1.12 5.71 1.36
II-SDHB**
29
Actin
42

89

Figure 5E

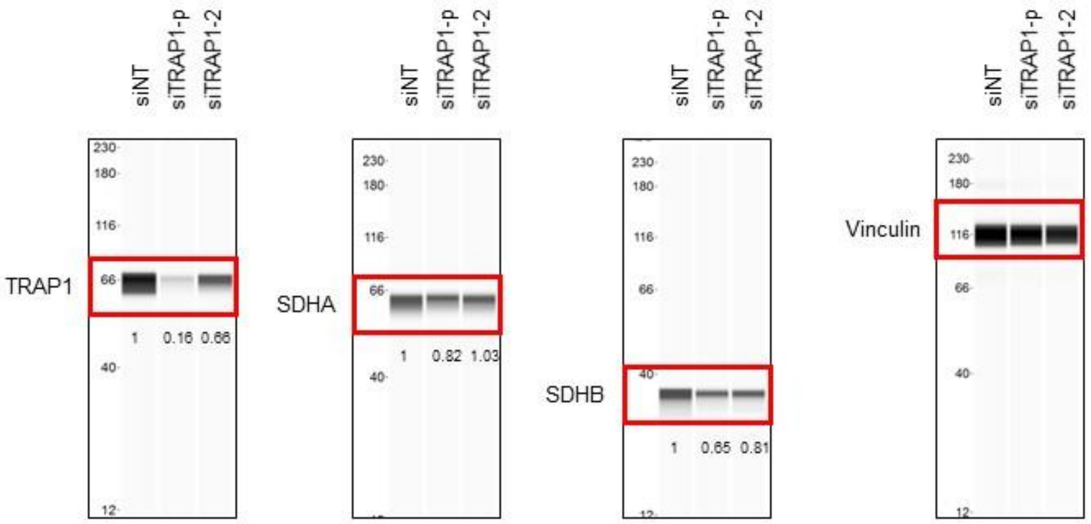
siNT
siTRAP1-p
siTRAP1-2
TRAP1
1 0.16 0.66
SDHA
1 0.82 1.03
SDHB
1 0.65 0.81
Vinculin
230
180
116
66
40
12

Figure S4C

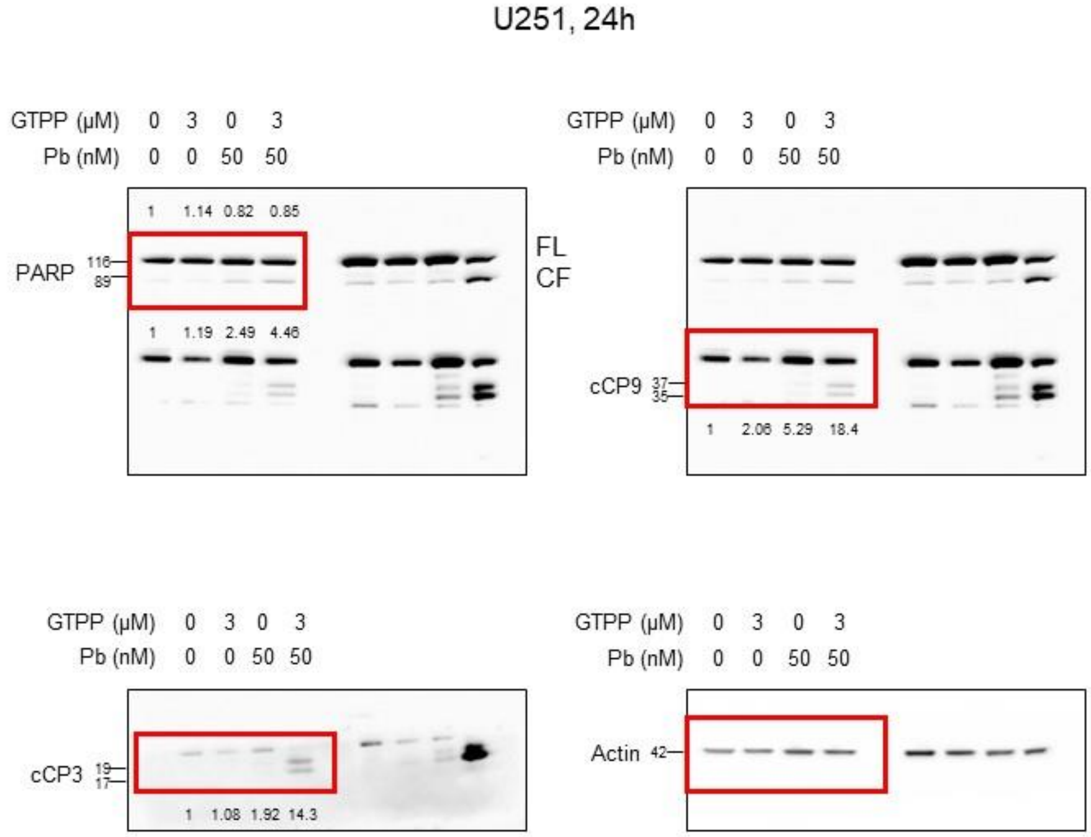

91

Figure S4C

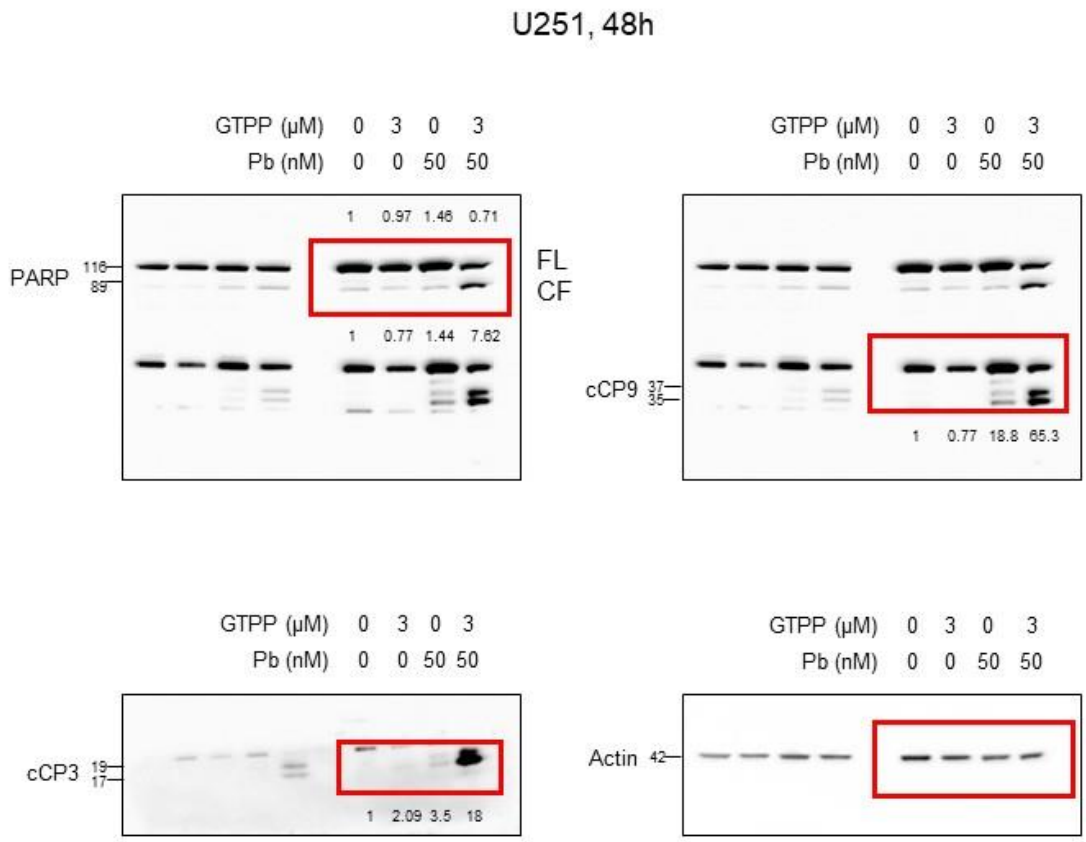
U251, 48h
GTPP (μM) 0 3 0 3
Pb (nM) 0 0 50 50
1 0.97 1.46 0.71
PARP
116
89
FL
CF
1 0.77 1.44 7.62
cCP9
37
35
1 0.77 18.8 65.3
cCP3
19
17
1 2.09 3.5 18
Actin
42

Figure S4D

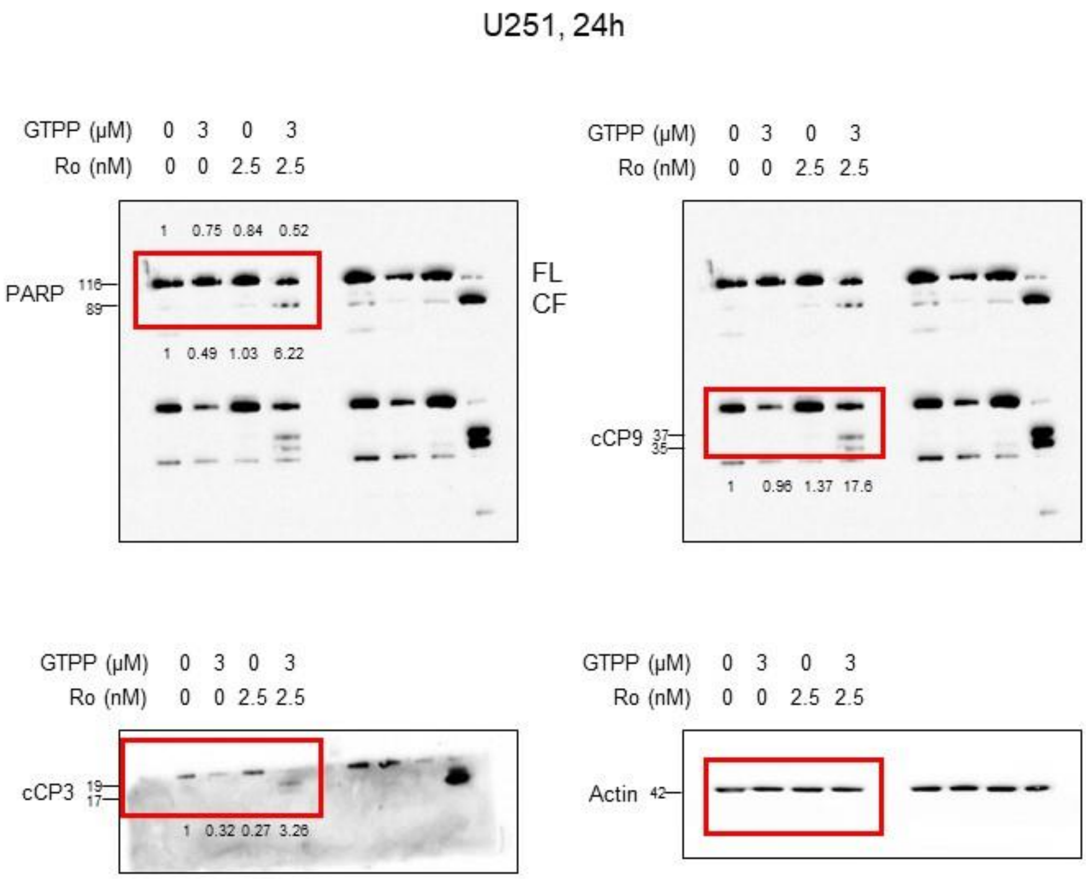

93

Figure S4D

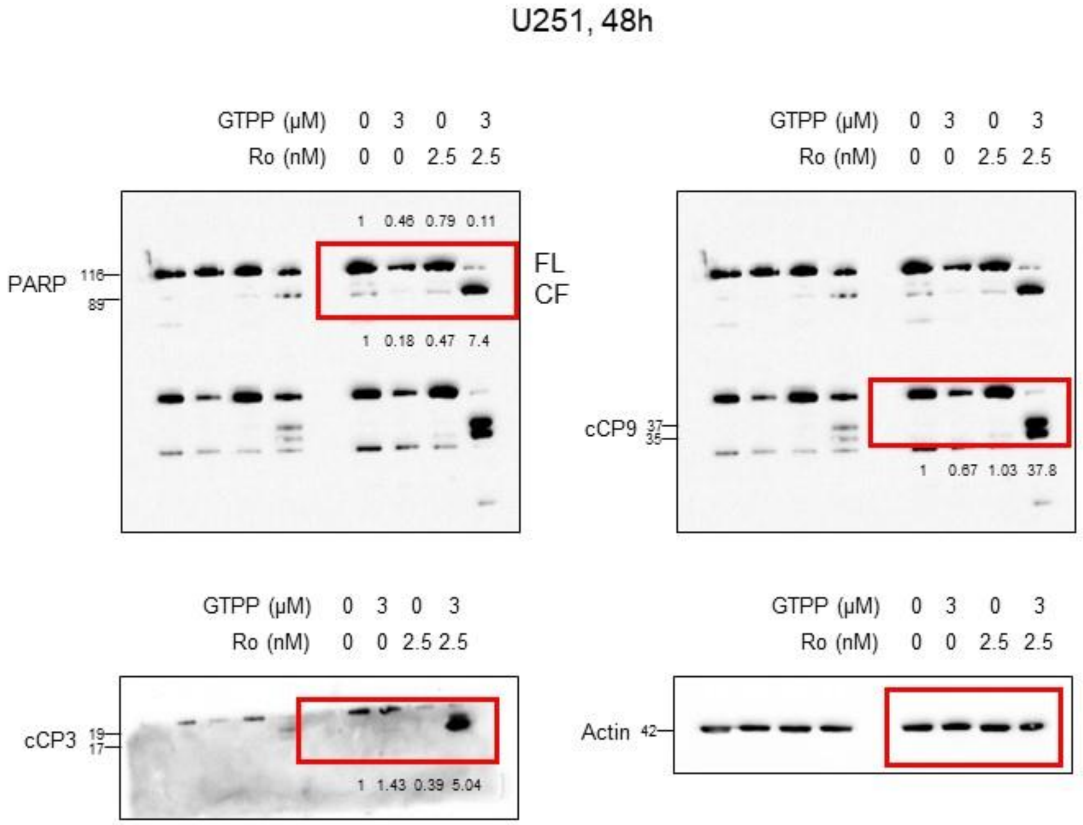
U251, 48h
GTPP (μM) 0 3 0 3
Ro (nM) 0 0 2.5 2.5
PARP
116
89
1 0.46 0.79 0.11
FL
CF
1 0.18 0.47 7.4
cCP9
37
35
1 0.67 1.03 37.8
cCP3
19
17
1 1.43 0.39 5.04
Actin
42

Figure S5B

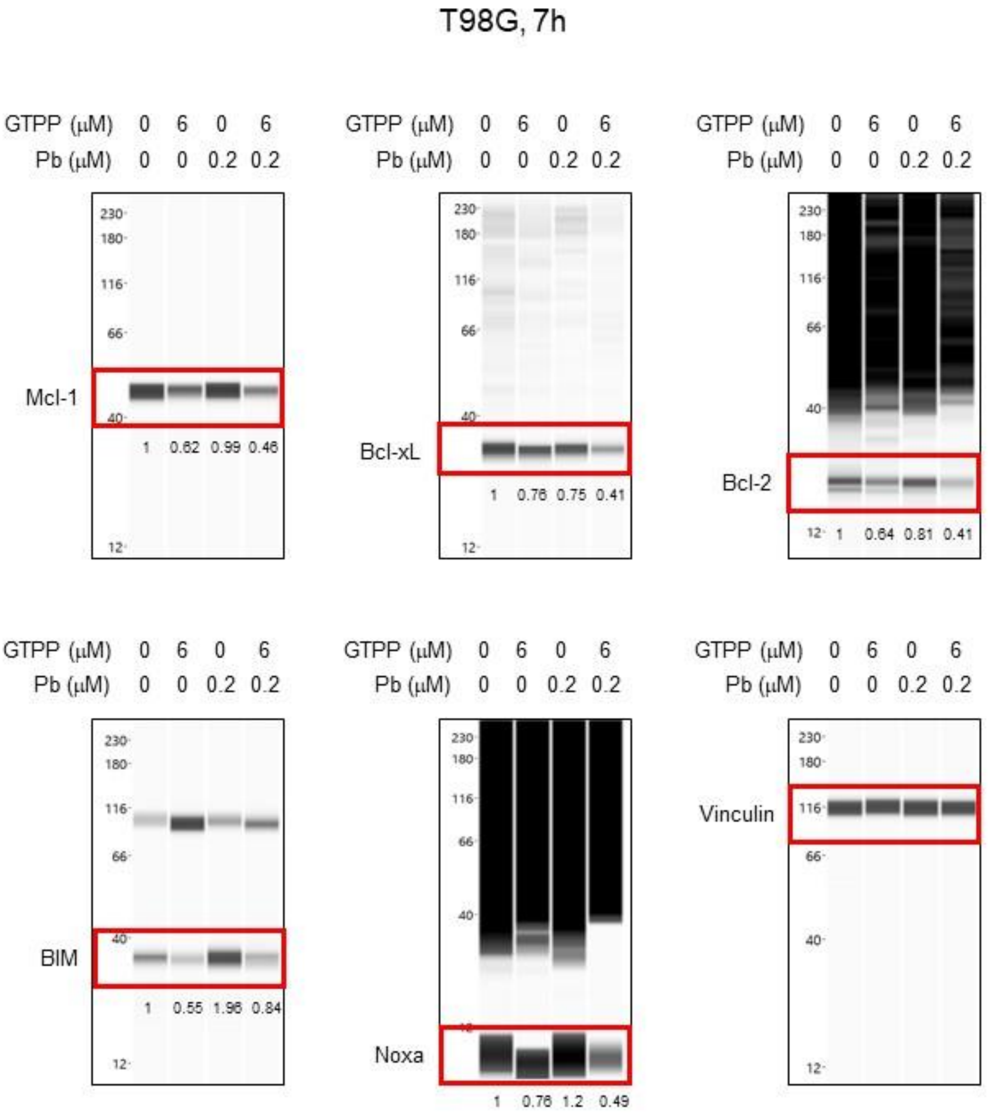
T98G, 7h
GTPP (μM) 0 6 0 6
Pb (μM) 0 0 0.2 0.2
Mcl-1
1 0.62 0.99 0.46
Bcl-xL
1 0.76 0.75 0.41
Bcl-2
1 0.64 0.81 0.41
BIM
1 0.55 1.96 0.84
Noxa
1 0.76 1.2 0.49
Vinculin

Figure S5B

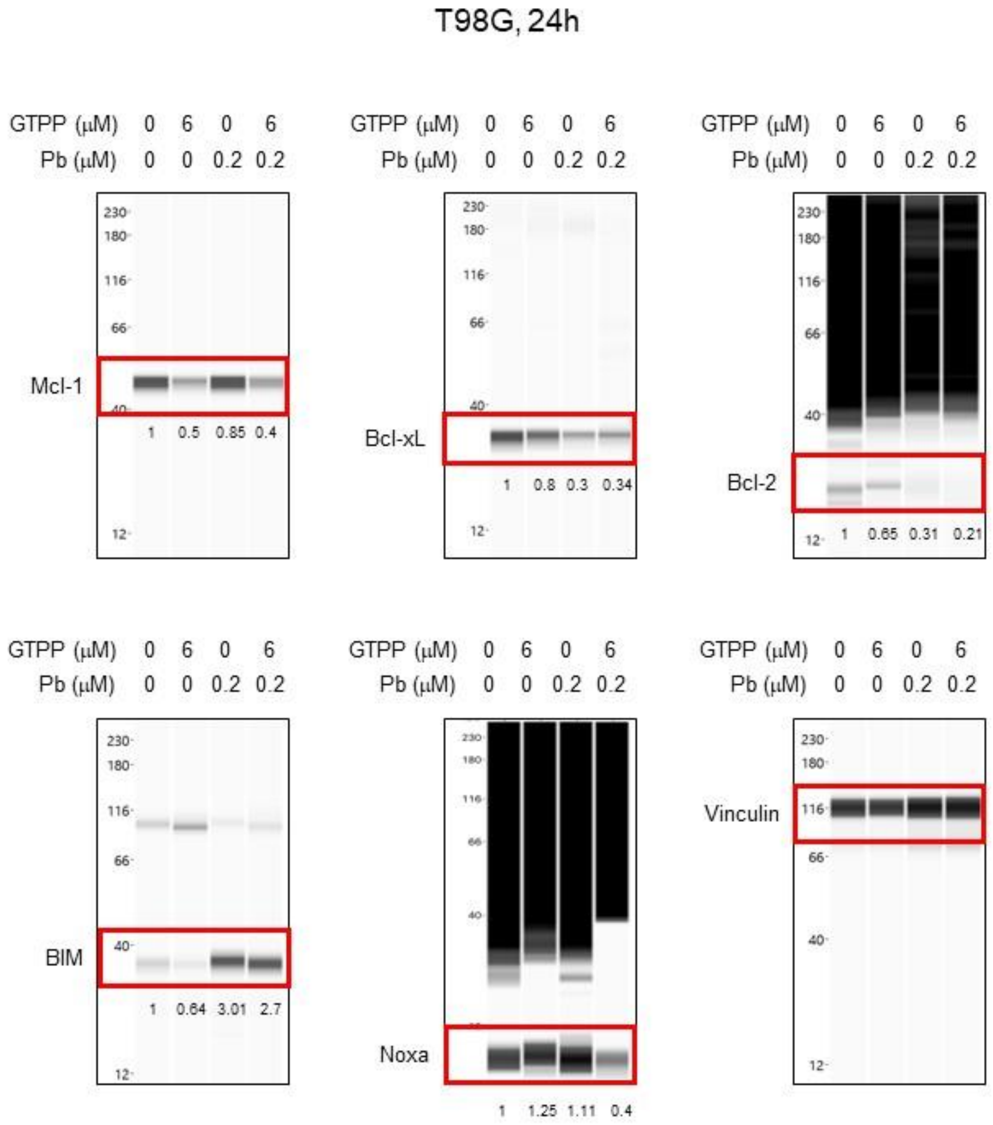
T98G, 24h
GTPP (μM) 0 6 0 6
Pb (μM) 0 0 0.2 0.2
Mcl-1
1 0.5 0.85 0.4
Bcl-xL
1 0.8 0.3 0.34
Bcl-2
1 0.65 0.31 0.21
BIM
1 0.64 3.01 2.7
Noxa
1 1.25 1.11 0.4
Vinculin
230
180
116
66
40
12

Figure S5D

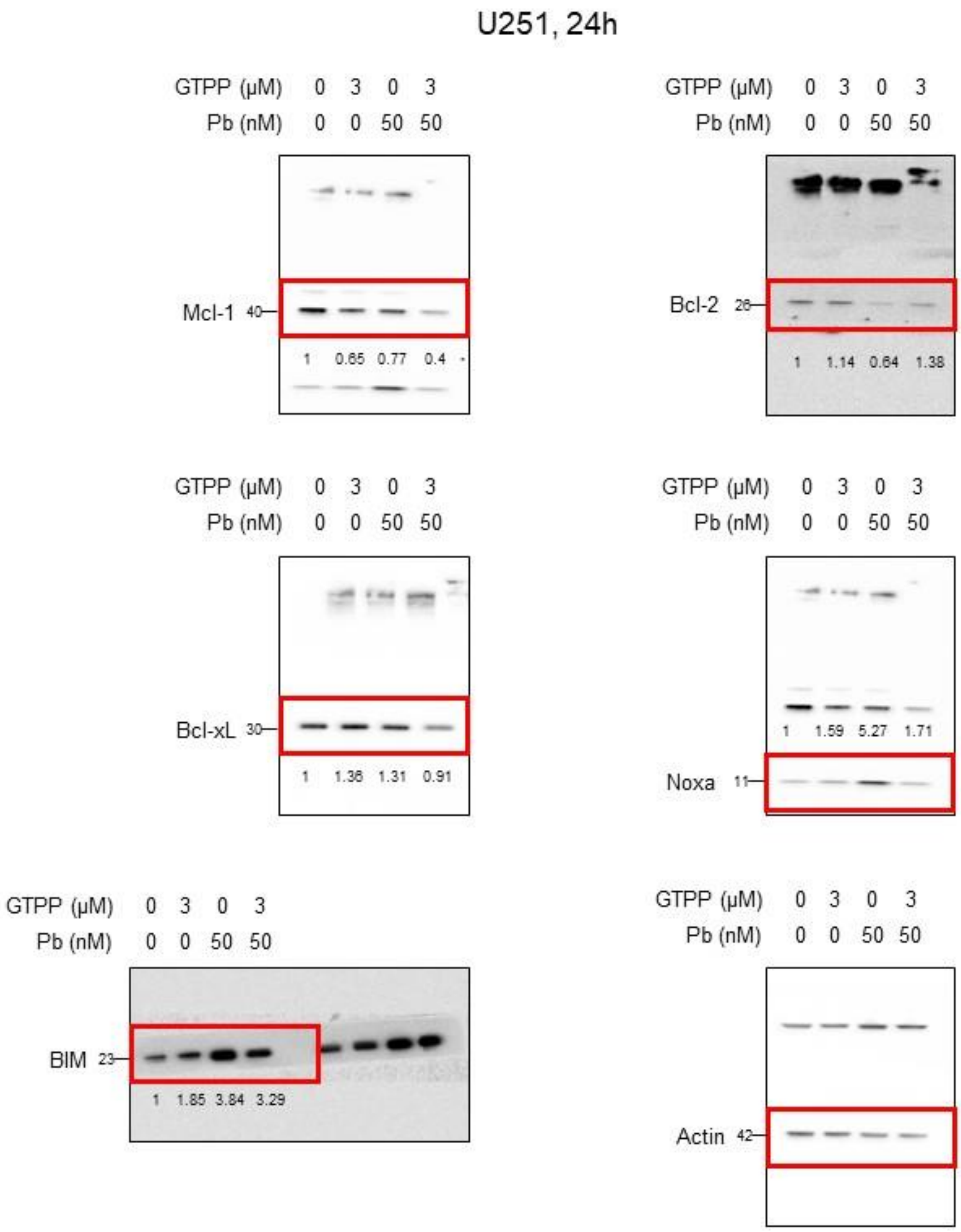
U251, 24h
GTPP (µM) 0 3 0 3
Pb (nM) 0 0 50 50
Mcl-1 40
1 0.65 0.77 0.4
Bcl-2 26
1 1.14 0.64 1.38
Bcl-xL 30
1 1.36 1.31 0.91
1 1.59 5.27 1.71
Noxa 11
BIM 23
1 1.85 3.84 3.29
Actin 42

Figure S4D

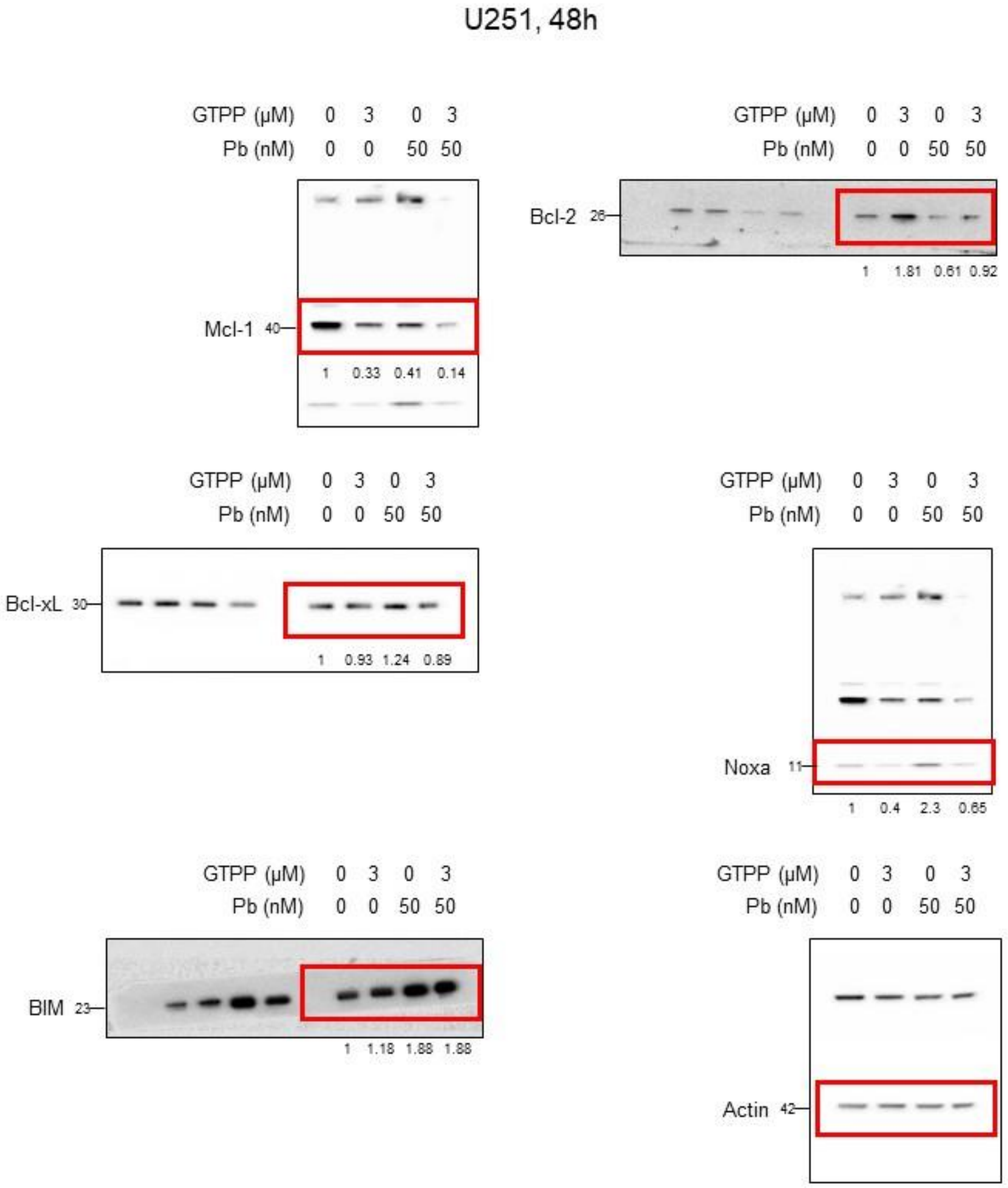

98

Figure S5E

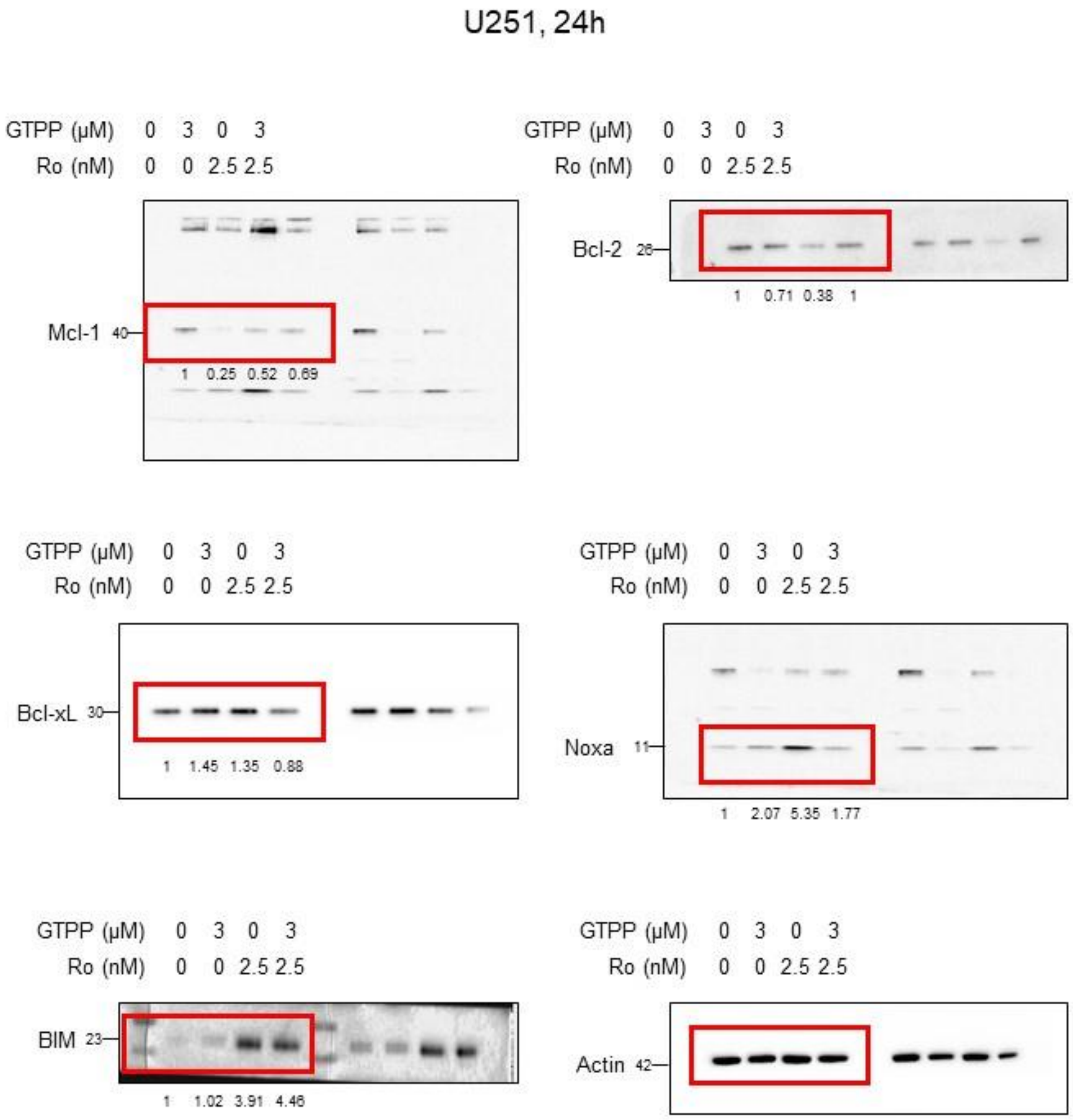
U251, 24h
GTPP (μM) 0 3 0 3
Ro (nM) 0 0 2.5 2.5
Mcl-1 40
1 0.25 0.52 0.69
Bcl-2 26
1 0.71 0.38 1
Bcl-xL 30
1 1.45 1.35 0.88
Noxa 11
1 2.07 5.35 1.77
BIM 23
1 1.02 3.91 4.46
Actin 42

Figure S5E

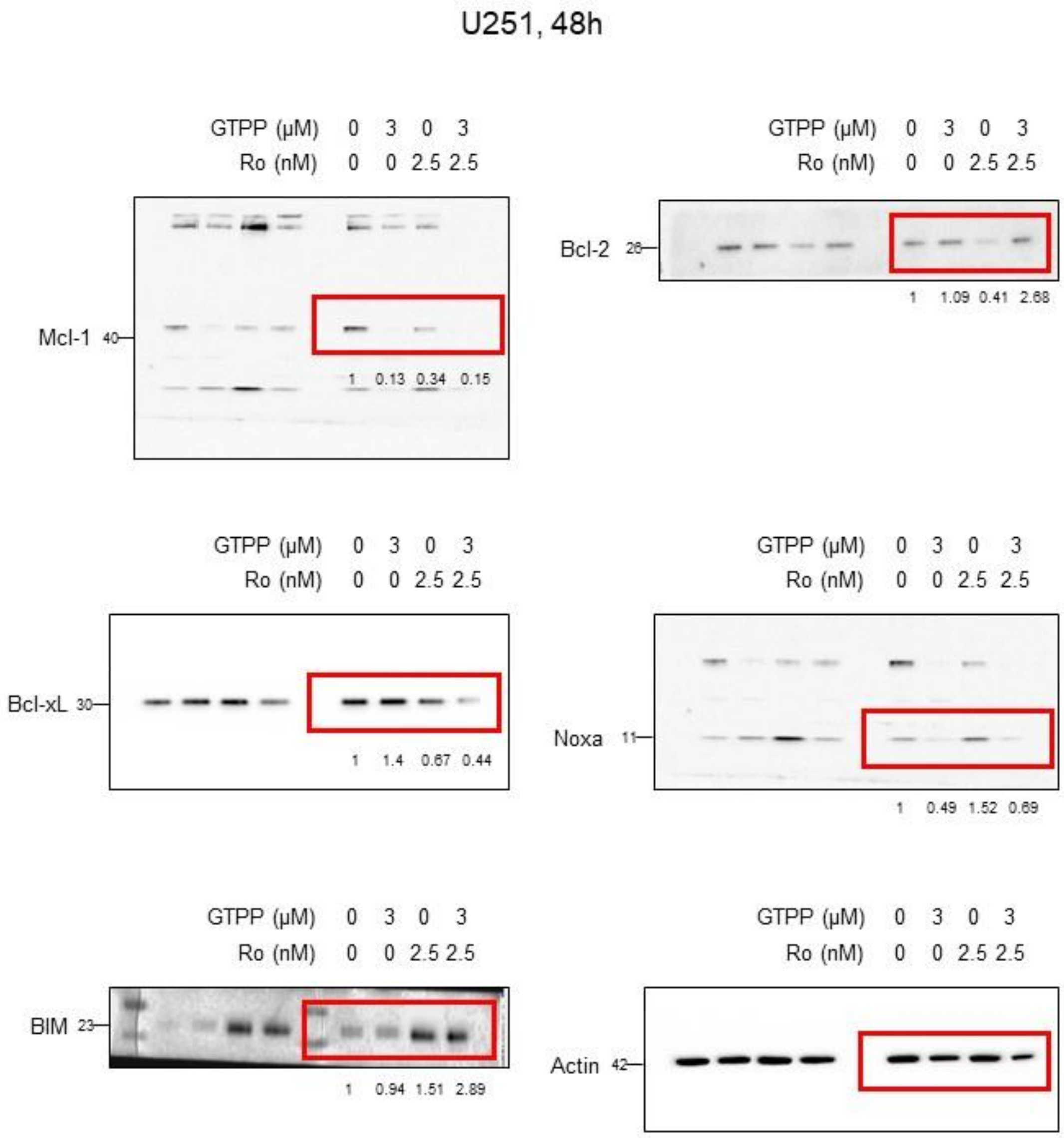

100

Figure S6B

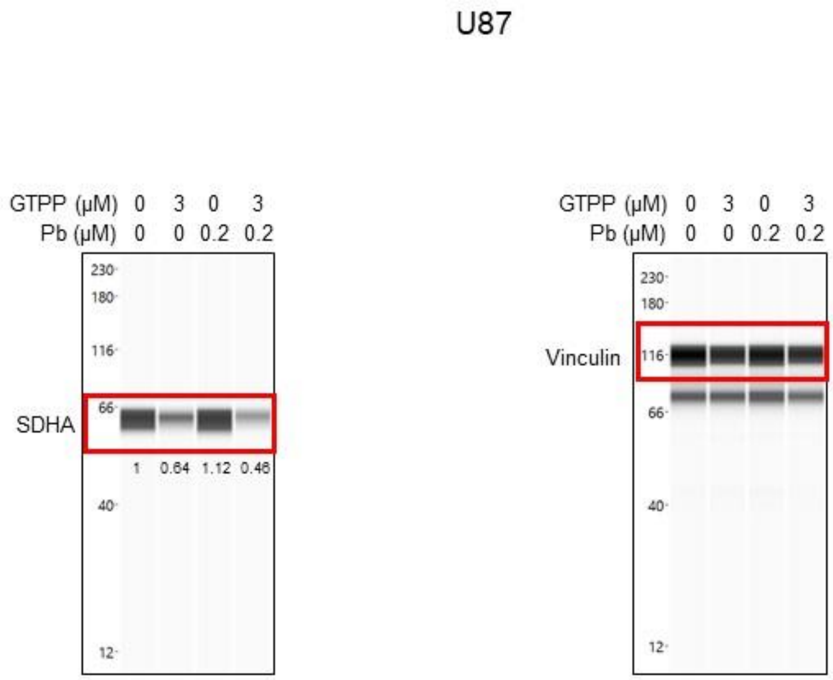

101

Figure S6B

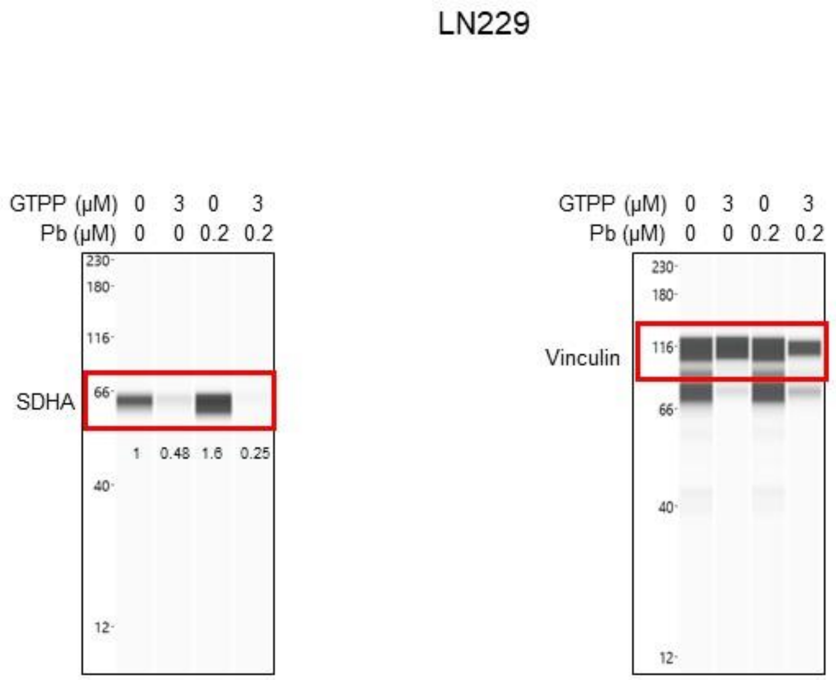
LN229
GTPP (μM) 0 3 0 3
Pb (μM) 0 0 0.2 0.2
230
180
116
66
SDHA
1 0.48 1.6 0.25
40
12
GTPP (μM) 0 3 0 3
Pb (μM) 0 0 0.2 0.2
230
180
116
Vinculin
66
40
12
